# Association between Socio-Demographic Factors and Owners’ Beliefs and Attitudes to Pet Cats Fundamental Dietary and Physical Exercise Needs, in City of Belfast

**DOI:** 10.3390/ani12192645

**Published:** 2022-10-01

**Authors:** Violetta Naughton, Teresa Grzelak, Maria S. Mulhern, Charlotte R. Moffett, Patrick J. Naughton

**Affiliations:** 1 School of Biomedical Sciences, University of Ulster, Cromore Road, Coleraine BT52 1SA, Northern Ireland, UK; 2 Department of Physiology, Poznan University of Medical Sciences, 61-701 Poznan, Poland

**Keywords:** pet cat owners, pet cat welfare, pet cat feeding, pet cat exercise, pet cat body condition

## Abstract

**Simple Summary:**

Pet cats need their humans for shelter, food, water, and company while, humans are responsible for providing such standard of care so as to ensure good health and welfare of their animals. This study investigated if owners’ beliefs and perceptions of pet cats’ needs and selected behaviours are associated with socio-demographic factors, in order to assess standards of fundamental care provided to animals. We found that selected socio-demographic factors, e.g., owner’s occupation or gender, are significantly associated with the owner’s beliefs/opinions and practices of care provided to their pet cats, e.g., type and amount of food or exercise provided to pet cats. The results of this study indicate that some owners may not have sufficient knowledge to provide an appropriate level of care to their cats and thus additional training in fundamental care for non-professionals (as opposed to veterinary and animal science professionals) would benefit the animals.

**Abstract:**

A cross-sectional survey questionnaire was developed in-house to investigate pet cat owners’ beliefs and attitudes related to the fundamental care of their pet cats. The questionnaire consisted of questions which were grouped into the following sections: (i) owners’ socio-demographics; (ii) cat(s) body weight and body condition monitoring; (iii) owners’ attitudes to cats’ dietary preferences, needs and satisfaction, (iv) owners’ perceptions of their cats’ physical exercise needs and satisfaction. The sample size of 376 was estimated to be required to represent the population of the given geographical location (Belfast, NI, UK). Hard copies of the questionnaires were distributed in January and February 2019 and in total 402 completed questionnaires were collected; questionnaires which included >20% of missing or incomprehensible responses were excluded from the database, resulting in 398 questionnaires being included in the final database. The study identified a number of socio-demographic factors associated with owners’ beliefs and attitudes that directly affect care provided to pet cats, e.g., the owner’s occupation has been identified as a factor associated with owner perception of certain cats’ behaviours, e.g., a cat brushing against the owner as food requests by their animal (Chi-Square 7.711 (df1), exact *p* = 0.006). Furthermore, most female respondents, aged 26–67 years and in an occupation not related to animals, reported selecting cat food based on their animal preferences (Chi-Square 10.332 (df1), exact *p* = 0.003). In contrast, female owners in animal and veterinary occupations were significantly more likely as compared to other respondents (Chi-Square 15.228 (df1), exact *p* < 0.001), to select cat food based on its perceived health benefit to the cat. Analysis of the respondents’ opinions of cats’ abilities to self-regulate physical activity showed that owners age was the main differentiating determinant, i.e., cat owners over 25 years old were significantly more likely than younger adults to believe that pet cats can regulate their own physical activity to keep healthy (Chi-Square 6.313 (df1), exact *p* = 0.025). Furthermore, respondents’ opinions of their cat’s ability to self-regulate feed intake were mainly associated with owner’s education level (Chi-Square 6.367 (df1), exact *p* = 0.036). The study results indicated that the attitude and beliefs behind the fundamental care practices provided to pet cats depends on particular demographic factors, especially owners’ education and occupation.

## 1. Introduction

Most recent surveys of pet animal ownership indicate that pet cats are the most popular pets in the European Union [1] and the second most popular pets in the UK [2], the USA [3] and Australia [4]. However, the literature also indicates that many pet cats worldwide are not provided with adequate care or appropriate living environments for good health and welfare or to prevent unwanted litters [5,6]. The study by Grigg et al. (2019) focusing on U.S.A. owners’ attitudes and care provided to pet cats has shown that many pet cats were receiving only minimal environmental enrichments, almost all pet cats were receiving minimal interactive play with humans, and some cats were positively punished for owner-perceived misbehaviour [7].

Recently, we have investigated more fundamental care practices of cat owners related to feeding/nutrition and physical activity provided to pet cats [8]. For example, we demonstrated that a significant majority of cat owners surveyed in 2019, in Northern Ireland did not use the Body Condition Scoring (BCS) method to assess their pet body condition and they did not weigh their pet [8]. Furthermore, the majority of owners fed their cats on animal demands or based on animal appetite, while not providing any play time to the animals [8]. It is acknowledged that daily food intake and daily exercise level are two principal components of energy balance that are both dependent on the pet owners’ correct assessment of their animal body weight and body condition. For example, overfeeding and/or lack of sufficient exercise may lead to animal obesity with reported obesity incidences for pet cats as high as 52% [9,10,11,12,13]. Inappropriate diet and/or feeding quantity combined with undetermined physical activity level, pose risks for the health and wellbeing of the pets, which in turn indicate a need to establish the potential drivers of such owners’ practices. Anecdotally, some cats always act hungry, which instigates feeding by the owners, or a common supposition is that pet cats can exercise themselves and thus they do not need to be exercised by the owners. To our knowledge however, there is no data on owners’ behavioural drivers, beliefs and attitudes that allow for a clear understanding of pet cat owners feeding or exercising practices. Therefore, in this cross-sectional descriptive study with the use of a survey questionnaire to collect primary data, we aimed to explore pet cat owners’ beliefs and attitudes of their cat’s dietary and physical needs, the animal’s ability to “self-regulate” their feed intake and their level of daily exercise. Furthermore, cat owners in this study were asked questions related to their socio-demographics to assess any potential association between owner’s attitudes and beliefs and socio-demographic factors. Our study was exploratory in nature hence not allowing for a study hypothesis, however we postulated that the identification of the drivers of the cat owners fundamental care practices, would allow for better understanding of determinants of common care provided to pet cats and in turn could potentially lead to the development of educational materials for pet cat owners to improve animal health and welfare.

## 2. Materials and Methods

An anonymous cross-sectional survey questionnaire was developed to investigate pet cat owners’ beliefs and attitudes related to the care of their pet cats. The draft questionnaire and the accompanying cover letter was developed and piloted in-house with random samples of cat owner volunteers (*n* = 14); and one-to-one interviews with volunteers’ feedback in relation to each part of the questionnaire were collected according to DeMaio et al. (1998) [14]. Subsequently, the draft questionnaire was amended, including rephrasing and reordering of some questions and the revised version was used for the current study. 

The final questionnaire consisted of questions which were grouped into the following sections: (i) owners’ demographics; (ii) cat(s)s body weight and body condition monitoring; (iii) owners’ attitudes to cats’ dietary preferences, needs and the drivers of shopping for cat food; (iv) owners’ perceptions of their cats needs and satisfaction. Table 1 presents a brief outline of the questionnaire used in the study. 

Ethical approval for the study was granted by the School of Biomedical Sciences Research Ethics Filter Committee, Ulster University (FCBMS-19-159). 

In order to minimize the influence of geographic and cultural differences on respondent data, the survey was made available only to responders residing in one geographical urban location, i.e., Belfast City Council district. The sample size of 376 completed questionnaires was estimated to be required to represent the population of the given geographical location. The sample size was based on data from the latest CENSUS (2011) [15] regarding the total number of households in Belfast City Council district vs. the regional pet population [16] and including 95% confidence interval and 5% margin of error. To account for missing/incomprehensible responses while assuring required representation, the target sample size was increased by 5% to 395 questionnaires to be collected. 

The following sampling approach was used to distribute and collect the questionnaires from the groups of interest, i.e.,: (i) pet cat owners, (ii) over 18 years of age, (iii) living in the selected geographical location. The hard copies of the questionnaires were distributed via local veterinary surgeries and pet supplies stores in January and February 2019. At the time of the distribution of the questionnaires, the prospective participants were asked to complete the questionnaires on site. However, the questionnaires were also supplied with pre-paid envelopes to those who were unable to complete the questionnaire at the time of the acceptance. Each questionnaire was accompanied with a consent letter that informed the respondents that only one questionnaire per household was required. The completion of questionnaires was treated as a consent to participate in the study and all responses were anonymous. Survey respondents received no monetary or any other compensation for completing the questionnaire. 

In total 402 completed questionnaires were collected. The data from all collected questionnaires were transferred into Excel (Microsoft Excel for Mac, v16.54, Volume License 2019, Microsoft, Dublin, RoI) and 10% of transferred data was blind-checked for transfer accuracy. Questionnaires which included >20% of missing or incomprehensible responses were excluded from the database and resulted in 398 questionnaires available to be included in the final database. Data in the final database were subsequently analysed with IBM SPSS for Windows (v25, IBM Inc., Armonk, NY, USA).

Frequency tables were created for all collected responses; the Chi-Square test and contingency test were used to assess potential associations between respondents’ demographic characteristics and their care practices. For the data subsets of a smaller sample size, Fisher Freeman Hilton exact test of independence was used (according to Cochran’s condition). We employed Cramer’s V coefficients for tables bigger than 2 × 2 tabulations to show the level of associations between respondents’ opinions and the sociodemographic ordinal variables [17]. In the case of both Spearman’s correlation analysis and Decision Trees CHAID algorithm, the questionnaire measurement was based on the Linkert scale. In the case of agree/disagree response options ranged from 1 (‘Strongly Disagree’) to 5 (‘Strongly Agree’); the midline at Likert score = 3 represented a neutral view (answers ‘Neutral’ and ‘Not sure’). For other response options ranged from 1 to 3 where 1 indicated ‘Not at all’, 2 indicated ‘Doubtfully’/‘Not sure’ and 3 indicated ‘Very much so’. Decision Trees CHAID (The Chi-Squared Automatic Interaction Detection) algorithm proposed by Kass (1980) was used to identify main determinants amongst all tested sociodemographic factors. Decision Trees CHAID operates using a series of merging, splitting, and stopping steps based on user-specified criteria, and was used for partitioning data into more homogeneous groups [18]. Chi-Square test for independence was performed to compare the categories of the predictor variable concerning the target variable with significance (alpha) set at 0.05; however, where needed we have used a splitting threshold that was greater than or equal to the merging threshold while the *p*-values for the merged categories were adjusted using a Bonferroni correction to control for type I error rate.

## 3. Results

### 3.1. Respondents’ Demographics

This study was based on the responses from 398 households owning pet cats. Half of the respondents (50.3%) belonged to 26–67 years old age group and were predominantly females (59.3%). The majority (85.2%) of respondents were in an occupation not related to animal sciences or veterinary medicine, in full time employment (46%) and completed a maximum secondary level education (50.8%). Table 2 shows full details of respondents’ demographics. 

### 3.2. Households’ Characteristics vs. Number of Pet Cats and Fundamental Care Provided to Cats

The households that participated in this study were categorised as three types, i.e.,: (i) single occupancy, multiple occupancy, either with (ii) only adult occupants, or (iii) adults with children. The households with adult occupants only comprised 46.2% of respondents, while single occupancy and adults with children households were represented by 24.6% and 29.1%, respectively. Table 3 shows full details of household characteristics. 

The majority of the households (64.3%) reported owning one pet cat. Household type did not influence the number of cats owned (Chi-Square 3.511 (df4), exact *p* = 0.476). Likewise, household type was not related to the frequency of monitoring cats body weight (Chi-Square 5.868 (df4), exact *p* = 0.208) or frequency of an assessment of body condition with Body Scoring System (Chi-Square 4.456 (df8), exact *p* = 0.814).

However, the type of multiple occupancy households, i.e., adults only vs. adults with children did have a significant effect on the number of people involved in feeding cat(s) (Chi-Square 63.171 (df5), *p* < 0.0001) as well as the number of people playing with cat(s) (Chi-Square 71.922 (df5), *p* < 0.0001), i.e., in both cases (feeding and playing) more people were involved in care of animals in households occupied by adults only (see Table 4 for details). 

### 3.3. Animals Living Environment and Fundamental Management Practices

#### 3.3.1. Living Environment Provided to Cats

The majority of the respondents (64.8%) indicated that their cats live indoors with free access to the outdoors (Table 5). Those not professionally involved with animals or veterinary medicine were significantly more likely to report that their cats live indoors with free access to the outdoors (Chi-Square 14.336 (df2), exact *p* = 0.001).

#### 3.3.2. Rationale behind Daily Feeding Amount

The responses to the question regarding the rationale of daily quantity of feed fed to their cat(s) varied but the most frequent reasons indicated by the respondents were either “feeding according to cat appetite” or “following instructions on food packaging “(30.4% of all respondents, for each reason). The responses to this question were however associated with owner’s gender, occupation, and employment (see Table 5 for details); further CHAID decision tree analysis showed that owner’s occupation was the main determinant of daily feeding quantity, i.e., those not professionally involved with animals or veterinary medicine were significantly more likely to feed their cats based on perceived animal appetite (Chi-Square 17.297 (df5), exact *p* = 0.004). 

#### 3.3.3. Frequency of Monitoring Animal Body Weight

When asked about the frequency of animal’s body weight monitoring, nearly half of all respondents (48%) declared that they do not weigh/monitor body weight of their cats. The responses to this question were associated with the respondents’ gender, occupation, and education (see Table 5 for details). However, further CHAID decision tree analysis showed that the owner’s occupation was the main determinant of responses to this question, i.e., those not professionally involved with animal or veterinary medicine were significantly more likely not to monitor weight of their cats (Chi-Square 34.613 (df2), exact *p* < 0.001). Furthermore, within this respondent group, pet cat owners with an education classified as level 5 or lower by the International Standard Classification of Education (ISCED) 2011 were significantly more likely not to monitor the weight of their cat(s) compared to respondents with a higher educational level (Chi-Square 10.319 (df2), exact *p* = 0.017).

#### 3.3.4. Body Condition Score (BCS): Knowledge and Use

The majority of the pet cat owners surveyed (56.0%; see also Table 5), indicated that they do not know and do not use the BCS. Those not professionally involved with animals or veterinary medicine were statistically significantly more likely not to know and so not use BCS (Chi-Square 62.503 (df4), exact *p* < 0.0001). Additionally, female respondents more often admitted that they do not know and do not use BCS (Chi-Square 12.050 (df3), exact *p* = 0.014) compared to male respondents. 

### 3.4. Owners’ Attitudes to Cats’ Dietary Preferences and Needs, and the Drivers of Shopping for Cat Food

**Figure 1:**
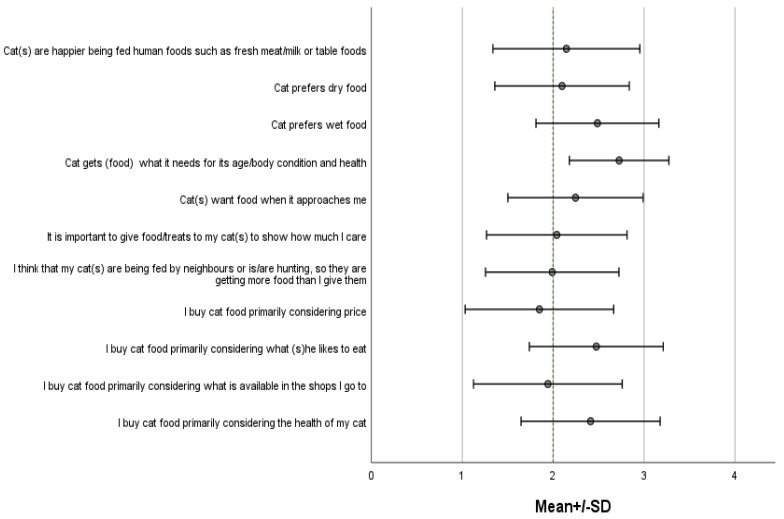
Owners attitude to cats dietary preferences, needs and the drivers of shopping for cat food. Response options ranged from 1 to 3 where 1 indicated ‘Not at all’, 2 indicated ‘Doubtfully’/‘Not sure’, 3 indicated ‘Very much so’.

An overview of the responses to the questions related to owner’s attitude to cat’s dietary preferences, needs and the drivers of shopping for cat food is presented in Figure 1.

#### 3.4.1. Owners Perception of Their Animal(s) Food Preferences

The majority of respondents (41%) believed their cats “are happier being fed human foods such as fresh meat or milk or table foods”. Further CHAID analysis showed that such opinion was most apparent for those in full-time education and others staying at home (unemployed or retired), (Chi-Square 7.739 (df2), exact *p* = 0.017) compared to other respondents (see Table 6 for details). 

The responses to a question about owners’ views on their cats’ contentment when being fed dry cat food, were varied (see Table 6 for details). Furthermore, the CHAID analysis showed the lack of a split decision tree in responses to this question and thus no sociodemographic determinants were identified. 

On the other hand, the majority of respondents (59%) declared that their cats are more content when being fed wet cat food. Further CHAID analysis showed the owners occupation was significantly associated with their views, i.e., the respondents in occupations not related to animal or vet sciences/medicine were significantly more likely (Chi-Square 4.180 (df1), exact *p* = 0.042) than others to agree that their cats are more content to be fed wet cat food. Refer to Table 6 for details.

#### 3.4.2. Owners’ Perceptions of an Expression of Care towards Their Animal(s)

The overall distribution of responses to the question of a feeding related significance of specific behaviours displayed by their cats, e.g., approaching the owner with tail up or brushing against the owner showed that the majority of the respondents (43%) perceive such behaviours as a cat request to be fed. However, further CHAID analysis showed the significant association between the owner’s occupation and this perception (Chi-Square 7.711 (df1), exact *p* = 0.006), with 45% of those in professions not related to animal (39% of all respondents) perceiving the behaviours listed above as food requests by their cat. Refer to Table 6 for details.

The respondents’ beliefs regarding offering a food treat to their cats as an expression of their care were varied (see Table 6 for details). Furthermore, the CHAID analysis showed the lack of a split decision tree in responses to this question and thus no sociodemographic determinants were identified. Refer to Table 6 for details. 

#### 3.4.3. Owner’s Perceptions of Their Animal(s) Dietary Needs

Over three-quarters of all respondents (77.6%) believed that the diet of their cats is fulfilling its “health needs”. Further CHAID analysis showed owners occupation as the main determinant (Chi-Square 19.692 (df1), exact *p* < 0.001) of such opinion, with male owners in occupations related to animals less often (41.7%) declaring that their cat(s) receive what it needs for its age/body condition and health as compared to female owners in animal related professions (68.8%). Refer to Table 6 for details.

The responses to a question about owners suspecting their cats being fed by the neighbours were diverse (see Table 6 for details). Furthermore, the CHAID analysis showed the lack of a split decision tree in responses to this question and thus no sociodemographic determinants were identified. 

#### 3.4.4. Owners’ Motivations Regarding Their Choices of Purchasing Commercial Food for Their Cat(s)

In the case of cat food selection considering the cost of food or availability, CHAID analysis showed that education was the main determinant of the respondents opinions for both questions, i.e., the majority of cat owners with an educational level exceeding ISCED 2011 level 4 versus others disagreed with “I buy cat food primarily considering price” (Chi-Square 7.722 (df1), exact *p* = 0.017) and “I buy cat food primarily considering what is available in the shops I go to” (Chi-Square 10.956(df1), exact *p* = 0.003). See also Table 6.

The majority of respondents (62.1%) declared that they buy cat food based on their animal preferences. However, the significant effect of the owner gender and age, when also considering owner occupation was identified in relation to the selection of cat food based on the perceived animal food preferences, i.e., “I buy cat food primarily considering what (s)he likes to eat”, i.e., majority of female, 26–67 years of age respondents in an occupation not related to animals (3) declared to select the cat food based on their animal preferences (Chi-Square 10.332(df1), exact *p* = 0.003). Refer to Table 6 for details.

Over half of all respondents (58.3%) declared that they buy cat food considering the health of their cat. However, female owners in animal and veterinary occupations were significantly more likely (Chi-Square 15.228 (df1), exact *p* < 0.001) to select cat food based on its perceived health benefit compared to other respondents.

### 3.5. Owners Perception of Their Cats Needs and Satisfaction

**Figure 2:**
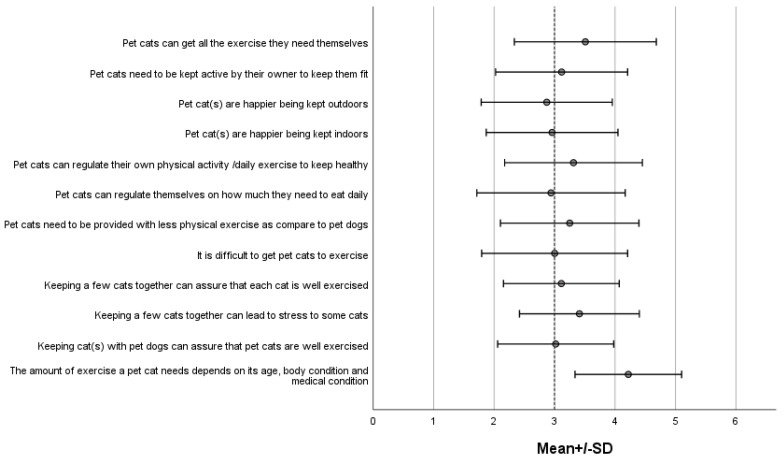
Owners perception of their cats needs and satisfaction. Response options ranged from 1 (“Strongly Disagree”) to 5 (“Strongly Agree”). The midline at Likert score = 3 represents a neutral view (answers “Neutral” and “Not sure”).

An overview of the responses to the questions related to owners’ perceptions/beliefs of their cat needs and satisfaction are presented in Figure 2. 

#### 3.5.1. Owners’ Perceptions of Their Animal’s Ability to Self-Regulate

CHAID analysis showed that occupation was the main factor differentiating pet cat owners’ opinions of pet cat’s ability to self-regulate their physical activity. Respondents in the occupation group not related to veterinary and animal science were significantly more likely (Chi-Square 8.535 (df1), exact *p* = 0.004) than the others to agree that “Pet cats can get all the exercise they need themselves”. Furthermore, CHAID analysis of the opinions of cats’ abilities to exercise to keep healthy showed that owners age was the main differentiating determinant here, i.e., cat owners over 25 years old were significantly more likely (Chi-Square 6.313 (df1), exact *p* = 0.025) than younger adults to believe that “Pet cats can regulate their own physical activity/daily exercise to keep healthy”. Refer to Table 7 for details.

Respondents opinions of cat’s ability to self-regulate feed intake were associated with owners education and this factor was confirmed as the main determinant by CHAID analysis, i.e., majority of cat owners with an educational level exceeding ISCED 2011 level 4 (Chi-Square 6.367 (df1), exact *p* = 0.036) versus others disagreed with the statement “Pet cats can regulate themselves on how much they need to eat daily”. The overall distribution of the responses is shown in Table 7. 

#### 3.5.2. Owners’ Perceptions of Cat’s Physical Activity Needs

When asked if cats owners need to keep their cats active, the respondents in veterinary and animal science occupations were significantly more likely (Chi-Square 6.122 (df1), exact *p* = 0.014) than the others to believe that “Pet cats need to be kept active by their owner to keep them fit”. The overall distribution of the responses is shown in Table 7.

The main factor differentiating responses to questions “Pet cats need to be provided with less physical exercise as compared to pet dog” and “It is difficult to get pet cats to exercise” was respondent’s employment status. Unemployed, retired, or those otherwise staying at home all the time were more likely (Chi-Square 10.710 (df1), exact *p* = 0.003) than others to be in favour of the opinion that, “Pet cats need to be provided with less physical exercise as compared to pet dogs” while the part-time employed owners were more likely (Chi-Square 6.502 (df1), exact *p* = 0.033) than the other groups to disagree with the opinion that “It is difficult to get pet cats to exercise”. Refer to Table 7 for details.

The result of Decision Trees CHAID showed that the response of cat owners with an educational level not exceeding ISCED 2011 level 6—was most consistent (Chi-Square 8.701 (df2), exact *p* = 0.001) with the opinion “The amount of exercise a pet cat needs depends on its age, body condition, and medical condition”. Notably, the respondents who completed ISCED 2011 level 1–4 were of this opinion more often (Chi-Square 5.180 (df1), exact *p* = 0.048), in particular male respondents and those who did not indicate their gender than other groups of respondents. 

#### 3.5.3. Owners’ Perceptions of Selected Environmental Factors Effects on Their Cats

Decision Trees CHAID analysis showed that the respondents in the occupation not related to veterinary and animal science and aged 67+ years were significantly more likely (Chi-Square 5.814 (df1), exact *p* = 0.033) to agree with the affirmative opinion on the issue “Keeping a few cats together can assure that each cat is well exercised”. On the other hand, all cat owners in veterinary and animal science occupations were significantly more likely to disagree with this view (Chi-Square 5.814 (df1), exact *p* = 0.033). The overall distribution of the responses is shown in Table 7.

## 4. Discussion

Within this study we surveyed 398 households owning pet cats. The surveyed households were either occupied by adult persons (one or more) or families with children. Our results have shown a significant effect of type of household on human involvement with pet cats, i.e., more people were involved in care of pet cats (feeding and playing) in households occupied by adults only as compared to the households occupied by families with children. A published report from Australia [4] showed that cat owners report spending an average of 4.2 h daily with their cats. However, to our knowledge there are no published data on the effects of type of household on time spent with cats or the number of people providing care to pet cats. Our results would indicate that both factors (the owners age and type of household) were directly associated with care provided to pet cats, thus further studies are required to explore both factors with the potential implications for cats’ health and welfare.

The majority of our respondents reported to own one cat regardless of the household type. These results are similar to our previous study from the whole province of Northern Ireland [8]. Recent data from Germany [19] also showed that owning one cat is most frequent in Germany. It is acknowledged that pet cats are not well adapted to living in close proximity to each other [20]. Furthermore, Mertens (1991) work has shown that singly kept pet cats had more playtime and other interaction with their owners as compared to group-living pet cats [21]. Podberscek et al. (1991) also reported that pet cats tend to play with their owners or alone rather than with other cats [22]. Although, pet cats living in groups over a period of time develop social organisation and present various social interactions [23,24], harmonious group-living of pet cats can only be successfully achieved if the environmental conditions are optimal (especially in relation to rest and retreat spaces) [25]. The results of this study showed that veterinary and animal science professionals are aware of behavioural characteristics of pet cats considering group-living, while non-professionals are not, e.g., they believe that group-living can assure that the cats are “well exercised”. 

The majority of our respondents indicated that their cats live indoors with free access to outdoors. Access to outdoors can be used as a proxy for activity levels in pet cats. However, the level of outdoor activity of domestic cats depends on the home location and the neighbourhood, as it has been shown that home ranges of pet cats are larger in rural sites compared to urban sites [26,27]. Furthermore, both home ranges and spatial movement of pet cats are determined by the density of cats in the area [28]. On the other hand, literature indicates that indoor pet cats interact more with their owners as compared to pet cats kept outdoors [29]. Since we did not investigate the rationale behind allowing pet cats outdoor access, it can be only be speculated that the owners are selecting more welfare-friendly living conditions for their pet cats at the expense of the interaction time with their pets, intentionally or not.

With regard to physical activity provided to pet cats by their owners, the professional (veterinary or animal sciences) respondents of this study declared that pet cats need to be kept active by their owners, while others believed that pet cats can self-regulate their physical activity level. Interestingly, when we asked the respondents of this study about exercising pet cats compared to pet dogs, those staying/working from home all of the time were of the opinion that cats needed less exercise than pet dogs as compared to owners from other socio-economic categories. Pet cats spend a large proportion of their, at-home daily time either resting or sleeping, thus it could be speculated that those owners who spend most of their time at home perceive that their cats are perhaps “tired” and do not require exercise. 

Our study found that the majority of owners who are not veterinarians/in animal science related occupations do not monitor their animal body weight, and are not familiar and thus do not use the body condition scoring, and feed their animals based on animal’s appetite, on animal demand and the perceived cat food preferences. The latter was particular to female adult cat owners in this study. Notably, this study also showed that the respondents of an educational ISCED 2011 level 4 and below believed that pet cats can self-regulate their food intake. Previously published studies have shown that ad libitum feeding, free choice feeding or high feeding frequency, in addition to feeding of food treats are associated with obesity in cats [11,30]. Furthermore, it has been previously shown that majority of cat owners do not follow any advice or feeding instructions and feed their cats “until it stops eating” [8,11]. Furthermore, this study also showed that cat owners who are not veterinarians or involved in animal sciences, were misinterpreting certain social behaviours of cats, e.g., vertical tail/tail up [31,32] as food soliciting behaviours. Such behaviours are social behaviours indicating deference in social status, which can certainly happen around food but are not always food soliciting. Thus, it could be tentatively suggested that the non-professional respondents of this study due to their beliefs and reported feeding management of their cats are exposing their animals to obesity and associated diseases. On reflection, appropriate animal care is based on a sound knowledge of animal biology, for example, one needs to assess animal body condition and body weight, and account for the level of exercise as well as animal physiological status to provide correct amount/composition and frequency of daily feed to assure that animals needs are met. Since those who are in veterinary (or animal sciences) professions are educated in various aspects of animal biology/management/disease prevention, etc. they are more likely to be able to provide appropriate care to their pet cats.

In this study, we have not investigated actual animal body condition, as the literature indicates that pet cat owners tend to underestimate their cat body condition. For example, Öhlund et al. (2018) have shown that 45% of pet cats when assessed by trained personnel were categorised as obese, while only 22% of the pet cats were reported as obese by their owners [33]. Furthermore, Kienzle et al. (2006) have shown that pet cat owners were less aware of the overweight problem of their pets than dog owners [34]. A recent study by Forrest et al. (2021) recognised incorrect owner perception of body condition as a potential risk factor for cats becoming overweight or obese [35]. Therefore, the investigations of the body conditions of animals in a clinical setting to assess incidences of pet cat obesity in any local population are warranted.

The results of analyses of demographic factors presented here need to be interpreted with caution. Although the required sample size for a given geographical location was attained, as with any questionnaire survey, reporting bias [36] needs to be taken into account. Furthermore, it needs to be emphasized that this exploratory descriptive study represents a specific geographical location hence further studies are required to explore the significance of sociodemographic factors in care provided to pet cats in other locations.

## 5. Conclusions

This study results showed that the attitude and beliefs behind the fundamental care practices provided to pet cats primarily depends on the socio-demographic factors. The belief that pet cats can regulate their physical activity/exercise level were significantly different in veterinary and animal sciences occupations as compared to those in any other occupation. This study has shown that pet owners professionally trained in veterinary and animal sciences, i.e., those with a knowledge of animal biology, were more aware of pet cats needs and care to assure animal health and welfare. It has been previously proposed that educating non-veterinary trained cat owners in fundamental aspects of pet care by veterinary professionals can potentially prevent ill health such as obesity and related conditions in pet cats [11,37]. Our results provide a basis for further understanding of existing attitudes and beliefs. It has been previously recognized elsewhere that any change in care provided to pets, if needed, must be based on such understanding to develop a successful program for change [38]. Thus, it could be postulated that training in fundamental care provided to pet cat owners, who are non-professionals would benefit the animals. However, whether such care training can change pet cat owners’ attitudes and beliefs and subsequent animal care would need to be assessed. 

## Figures and Tables

**Table 1:** An overview of the questionnaire used in the study.

Questionnaire Section	Data Collected
(i) Owners demographics	
	Age
	Gender
	Occupation
	Education
	Employment status
	Household type and number of persons living and involved with the care of pets
	Number of cats kept in a household
(ii) Cat(s) body weight and body condition monitoring by the owners	
	Daily quantity of feed fed to the animal
	Frequency of monitoring animal body weight
	Knowledge and use of Body Condition Score (BCS)
(iii) Owners attitude of cats dietary preferences, needs and the drivers of shopping for cat food ^1^	
	animal(s) food preferences (dry, wet, homemade)
	giving food as an expression of care towards their animal(s)
	animal(s) dietary needs (fulfilled or not)
	owners motivation behind purchasing commercial food for their cat(s)
(iv) Owners perception of their cats needs and satisfaction ^2^	
	Owners perception of their animal ability to self-regulate (feed intake and exercise)
	Owners perception of cats needs for physical activity
	Owners perception of their cats living environment (in/out, etc.)

^1^ Respondents were asked to state their agreement level using a Likert scale of 1–3, where 1 indicates ‘not at all’, 2 indicates ‘doubtfully’, 3 indicates ‘very much so’, and 4 indicates ‘not sure’. ^2^ Respondents were asked to state their agreement level using a Likert scale of 1–5 where 1 indicates ‘strongly agree’ to 5 ‘strongly disagree’, and 6 indicates ‘not sure’.

**Table 2:** Respondents’ demographics.

Characteristic		*n*	%
Age group			
	18–25	133	33.4
	26–67	200	50.3
	67+	65	16.3
	Total	398	100
Gender			
	Female	236	59.3
	Male	151	37.9
	Not disclosed	11	2.8
	Total	398	100
Occupation			
	Not related to veterinary or animal sci.	339	85.2
	Veterinary, and animal sci. related	59	14.8
	Total	398	100
Education			
	ISCED ^1^ 2011 level 1–4	202	50.8
	ISCED 2011 level 5–6	116	29.1
	ISCED 2011 level 7–8	54	13.6
	Not disclosed	26	6.5
	Total	398	100
Employment status			
	Full-time	183	46.0
	Part-time	84	21.1
	Full time education	37	9.3
	Unemployed, retired, or otherwise at home and not working	94	23.6
	Total	398	100

^1^ ISCED—International Standard Classification of Education; level 1–4: from primary to post-secondary; level 5–6: short-cycle tertiary education and Bachelor’s or equivalent; level 7–8: Masters or equivalent and Doctoral or equivalent.

**Table 3:** Households characteristics vs. number of cats owned.

Number of Cats	Household Type							
	Single		Adults Only		Adults with Children		Total	
	*n*	%	*n*	%	*n*	%	*n*	%
one	56	14.1	125	31.4	75	18.8	256	64.3
two	29	7.3	43	10.8	30	7.5	102	25.6
three or more	13	3.3	16	4.0	11	2.8	40	10.1
Total	98	24.6	184	46.2	116	29.1	398	100

**Table 4:** Household with multiple occupancy (*n* = 300) vs. number of people involved with cat(s) care.

		(A) Number of People Feeding Cat(s)			(B) Number of People Playing with Cat(s)		
		Household Type			Household Type		
Number of People Involved in Cat Care		Adults Only	Adults with Children	Total	Adults Only	Adults with Children	Total
none	*n*	0	1	1	7	6	13
	%	0	0.3	0.3	2.3	2	4.3
one	*n*	46	25	71	43	20	63
	%	15.3	8.3	23.7	14.3	6.7	21
two	*n*	63	35	98	57	27	84
	%	21	11.7	32.7	19	9	28
three or more	*n*	61	25	86	55	25	80
	%	20.3	8.3	28.7	18.3	8.3	26.7
not sure	*n*	13	26	39	15	34	49
	%	4.3	8.7	13	5	11.3	16.3
no answer provided	*n*	1	4	5	7	4	11
	%	0.3	1.3	1.7	2.3	1.3	3.7
Total	*n*	184	116	300	184	116	300
	%	61.3	38.7	100	61.3	38.7	100

**Table 5:** Animals living environment and fundamental management practices, including, feeding practices, monitoring animal body weight and knowledge, and use of Body Condition Score (BCS) reported by the cat owners in this study. A full set of cross-tabulated results is shown in Supplementary Materials (Table S1).

		Socio-Demographic Factors Evaluated in the Study				
	All Respondents	Effects of Age	Effects of Gender	Effects of Occupation	Effects of Education	Effects of Employment
	*n* (%)	Cramer’s V Coefficient (C’sV) ^1^ *p* Value ^2^				
(I) Living environment provided to cats						
indoors (no access outdoors)	106 (26.6)	C’sV = 0.095 *p* = 0.130	C’sV = 0.107 *p* = 0.060	C’sV = 0.190 *p* < 0.001	C’sV = 0.092 *p* = 0.350	C’sV = 0.121 *p* = 0.071
indoors with a free access to outdoors	258 (64.8)					
outdoors with an access to shelter	34 (8.5)					
Total	398 (100)					
(II) Rationale behind daily feeding amount						
fed according to cat appetite	121 (30.4)	C’sV = 0.136 *p* = 0.144	C’sV = 0.206 *p* < 0.001	C’sV = 0.208 *p* = 0.004	C’sV = 0.129 *p* = 0.172	C’sV = 0.158 *p* = 0.012
following the advice from another person (non-professional)	47 (11.8)					
following veterinary advice	69 (17.3)					
following instructions on food packaging	121 (30.4)					
based on visual inspection of the animal behaviour	38 (9.5)					
no answer provided	2 (0.5)					
Total	398 (100)					
(III) Frequency of monitoring animal body weight						
every 3–6 months	91 (22.9)	C’sV = 0.105 *p* = 0.067	C’sV = 0.122 *p* = 0.019	C’sV = 0.295 *p* < 0.001	C’sV = 0.156 *p* = 0.004	C’sV = 0.107 *p* = 0.164
once a year	116 (29.1)					
I do not monitor	191 (48.0)					
Total	398 (100)					
(IV) Body Condition Score (BCS): knowledge and use						
I know BCS and use it	65 (16.3)	C’sV = 0.144 *p* = 0.036	C’sV = 0.174 *p* = 0.002	C’sV = 0.396 *p* < 0.001	C’sV = 0.141 *p* = 0.021	C’sV = 0.135 *p* = 0.040
I know of BCS, but not sure how to use it	74 (18.6)					
I am vaguely familiar with BCS thus I do not use it	35 (8.8)					
I do not know and do not use BCS	224 (56.3)					
Total	398 (100)					

^1^ Cramer’s V coefficients (C’sV) show the level of associations between respondents’ opinions and the sociodemographic ordinal variables listed in the table columns. C’sV between 0.100–0.150 indicate moderate while C’sV between 0.151–0.250 indicate strong association [17]. ^2^ *p* values for Pearson’s chi-square tests or those for Fisher Freeman Hilton exact test for the data-subsets of smaller sample size according to Cochran condition.

**Table 6:** Owners attitude of cats dietary preferences, needs and the drivers of shopping for cat food. A full set of cross-tabulated results is shown in Supplementary Materials (Table S2).

						Socio-Demographic Factors Evaluated in the Study				
		Responses				Effects of Age	Effects of Gender	Effects of Occupation	Effects of Education	Effects of Employment
		Not at All	Doubt-Fully	Very Much So	Not Sure					
		[%]				Cramer’s V coefficient (C’sV) ^1^ *p* Value ^2^				
Owners perception of their animal(s) food preferences: I think that …										
1	… my cat(s) are happier being fed human foods such as fresh meat or milk or table foods	26.4	19.3	41.0	13.3	C’sV = 0.091 *p* = 0.763	C’sV = 0.071 *p* = 0.673	C’sV = 0.130 *p* = 0.077	C’sV = 0.161 *p* = 0.306	C’sV = 0.175 *p* = 0.184
2	… my cat(s) are happier being fed dry commercial food	22.9	33.2	32.7	11.3	C’sV = 0.153 *p* = 0.147	C’sV = 0.075 *p* = 0.895	C’sV = 0.105 *p* = 0.221	C’sV = 0.133 *p* = 0.617	C’sV = 0.161 *p* = 0.307
3	… my cat(s) are happier being fed wet commercial food	10.3	21.6	59.0	9.0	C’sV = 0.095 *p* = 0.311	C’sV = 0.081 *p* = 0.517	C’sV = 0.115 *p* = 0.150	C’sV = 0.168 *p* = 0.239	C’sV = 0.159 *p* = 0.329
Owners perception of an expression of care towards their animal(s): I think that…										
1	… my cat(s) want food when it approaches me, e.g., with tail up or brushing against me	18.3	21.1	43.0	17.6	C’sV = 0.094 *p* = 0.734	C’sV = 0.101 *p* = 0.225	C’sV = 0.143 *p* = 0.043	C’sV = 0.084 *p* = 0.499	C’sV = 0.093 *p* = 0.323
2	… it is important to give food/treats to my cat(s) to show how much I care	27.9	28.6	31.9	11.6	C’sV = 0.087 *p* = 0.427	C’sV = 0.108 *p* = 0.158	C’sV = 0.091 *p* = 0.351	C’sV = 0.102 *p* = 0.186	C’sV = 0.072 *p* = 0.720
Owners perception of their animal(s) dietary needs: I think that…										
1	… food wise, my cat(s) gets what it needs for its age/body condition and health	5.0	9.8	77.6	7.5	C’sV = 0.154 *p* = 0.138	C’sV = 0.246 *p* < 0.001	C’sV = 0.240 *p* < 0.001	C’sV = 0.166 *p* = 0.253	C’sV = 0.060 *p* = 0.886
2	… my cat(s) are being fed by neighbours or is/are hunting, so they are getting more food than I give them	27.4	25.6	26.4	20.6	C’sV = 0.094 *p* = 0.317	C’sV = 0.079 *p* = 0.540	C’sV = 0.099 *p* = 0.273	C’sV = 0.081 *p* = 0.550	C’sV = 0.128 *p* = 0.020
Owners motivation regarding their choices of purchasing commercial food for their cat(s): I buy cat food primarily …										
1	… considering price	42.0	24.1	26.9	7.0	C’sV = 0.094 *p* = 0.324	C’sV = 0.113 *p* = 0.115	C’sV = 0.048 *p* = 0.823	C’sV = 0.105 *p* = 0.161	C’sV = 0.072 *p* = 0.722
2	… considering what (s)he likes to eat	14.6	17.1	62.1	6.3	C’sV = 0.148 *p* = 0.008	C’sV = 0.244 *p* < 0.001	C’sV = 0.175 *p* = 0.006	C’sV = 0.146 *p* = 0.466	C’sV = 0.099 *p* = 0.915
3	… considering what is available in the shops I go to	36.4	28.6	30.7	4.3	C’sV = 0.051 *p* = 0.910	C’sV = 0.102 *p* = 0.222	C’sV = 0.125 *p* = 0.103	C’sV = 0.112 *p* = 0.096	C’sV = 0.116 *p* = 0.067
4	… considering the health of my cat	17.1	15.6	58.3	9.0	C’sV = 0.175 *p* < 0.001	C’sV = 0.202 *p* = 0.009	C’sV = 0.171 *p* = 0.007	C’sV = 0.134 *p* = 0.608	C’sV = 0.149 *p* = 0.466

^1^ Cramer’s V coefficients (C’sV) show the level of associations between respondents’ opinions and the sociodemographic ordinal variables listed in the table columns. C’sV between 0.100–0.150 indicate moderate while C’sV between 0.151–0.250 indicate strong association [17]. ^2^ *p* values for Pearson’s chi-square tests or those for Fisher Freeman Hilton exact test for the data-subsets of smaller sample size according to Cochran’s condition.

**Table 7:** Owners’ perceptions of their cats’ needs and satisfaction. A full set of cross-tabulated results is shown in Supplementary Materials (Table S3).

								Socio-Demographic Factors Evaluated in the Study				
		Responses ^1^						Effects of Age	Effects of Gender	Effects of Occupation	Effects of Education	Effects of Employment
		SA	A	N	SD	D	NS					
		[%]						Cramer’s V Coefficient (C’sV) ^2^ *p* Value ^3^				
Owners perception of their animal ability to self-regulate												
1	Pet cats can get all the exercise they need themselves	21.1	35.7	19.3	8.3	10.3	5.3	C’sV = 0.133 *p* = 0.714	C’sV = 0.149 *p* = 0.062	C’sV = 0.208 *p* = 0.004	C’sV = 0.108 *p* = 0.532	C’sV = 0.102 *p* = 0.652
2	Pet cats can regulate their own physical activity/daily exercise to keep healthy	12.3	38.7	23.1	9.8	12.3	3.8	C’sV = 0.138 *p* = 0.125	C’sV = 0.135 *p* = 0.688	C’sV = 0.176 *p* = 0.031	C’sV = 0.120 *p* = 0.310	C’sV = 0.119 *p* = 0.317
3	Pet cats can regulate themselves on how much they need to eat daily	7.5	32.7	16.8	17.1	19.3	6.5	C’sV = 0.118 *p* = 0.349	C’sV = 0.127 *p* = 0.232	C’sV = 0.137 *p* = 0.191	C’sV = 0.119 *p* = 0.317	C’sV = 0.102 *p* = 0.647
Owners perception of cats physical activity needs												
1	Pet cats need to be kept active by their owner to keep them fit	7.0	35.9	26.1	9.0	20.1	1.8	C’sV = 0.097 *p* = 0.676	C’sV = 0.128 *p* = 0.227	C’sV = 0.145 *p* = 0.126	C’sV = 0.126 *p* = 0.218	C’sV = 0.148 *p* = 0.036
2	Pet cats need to be provided with less physical exercise as compare to pet dogs	10.8	39.4	15.8	9.3	17.3	7.3	C’sV = 0.147 *p* = 0.072	C’sV = 0.081 *p* = 0.871	C’sV = 0.078 *p* = 0.790	C’sV = 0.130 *p* = 0.166	C’sV = 0.137 *p* = 0.097
3	It is difficult to get pet cats to exercise	11.6	26.6	19.3	11.6	26.4	4.5	C’sV = 0.103 *p* = 0.589	C’sV = 0.114 *p* = 0.413	C’sV = 0.074 *p* = 0.825	C’sV = 0.100 *p* = 0.675	C’sV = 0.129 *p* = 0.173
4	The amount of exercise a pet cat needs depends on its age, body condition and medical condition	43.7	41.2	6.0	2.0	2.5	4.5	C’sV = 0.118 *p* = 0.354	C’sV = 0.190 *p* = 0.001	C’sV = 0.169 *p* = 0.045	C’sV = 0. 164 *p* = 0.006	C’sV = 0.129 *p* = 0.176
Owners perception of selected environmental factors effects on their cats												
1	Keeping a few cats together can assure that each cat is well exercised	6.5	26.4	23.9	6.0	16.1	21.1	C’sV = 0.110 *p* = 0.475	C’sV = 0.155 *p* = 0.038	C’sV = 0.188 *p* = 0.015	C’sV = 0.188 *p* = 0.015	C’sV = 0.117 *p* = 0.364
2	Keeping a few cats together can lead to stress to some cats	11.3	38.2	17.6	6.0	7.5	19.3	C’sV = 0.092 *p* = 0.745	C’sV = 0.108 *p* = 0.510	C’sV = 0.087 *p* = 0.710	C’sV = 0.151 *p* = 0.026	C’sV = 0.097 *p* = 0.734
3	Keeping cat(s) with pet dogs can assure that pet cats are well exercised	6.8	19.8	24.9	6.8	17.8	23.9	C’sV = 0.134 *p* = 0.157	C’sV = 0.118 *p* = 0.348	C’sV = 0.240 *p* < 0.001	C’sV = 0.129 *p* = 0.173	C’sV = 0.121 *p* = 0.288
4	Pet cat(s) are happier being kept outdoors	5.5	23.4	32.2	13.1	21.1	4.8	C’sV = 0.188 *p* = 0.002	C’sV = 0.090 *p* = 0.774	C’sV = 0.169 *p* = 0.045	C’sV = 0.169 *p* = 0.004	C’sV = 0.160 *p* = 0.010
5	Pet cat(s) are happier being kept indoors	6.5	25.4	34.2	12.3	17.8	3.8	C’sV = 0.094 *p* = 0.715	C’sV = 0.077 *p* = 0.912	C’sV = 0.065 *p* = 0.891	C’sV = 0.104 *p* = 0.608	C’sV = 0.102 *p* = 0.647

^1^ SA = Strongly agree, A = Agree, N = Neutral, SA = Strongly disagree, D = Disagree, NS = Not sure. ^2^ Cramer’s V coefficients (C’sV) show the level of associations between respondents’ opinions and the sociodemographic ordinal variables listed in the table columns. C’sV between 0.100–0.150 indicate moderate while C’sV between 0.151–0.250 indicate strong association [17]. ^3^ *p* values for Pearson’s chi-square tests or those for Fisher Freeman Hilton exact test for the data-subsets of smaller sample size according to Cochran’s condition.

## Data Availability

Not applicable.

## Supplementary Materials

The following supporting information can be downloaded at: https://www.mdpi.com/article/10.3390/ani12192645/s1, Table S1, Table S2, Table S3—with cross-tabulated results supporting Table 5, Table 6 and Table 7 respectively).
