# Supplementary material for: Association between Socio-Demographic Factors and Owners’ Beliefs and Attitudes to Pet Cats Fundamental Dietary and Physical Exercise Needs, in City of Belfast
Source: Animals (Basel). 2022 Oct 1;12(19):2645. doi: 10.3390/ani12192645 (PMC9559292; doi:10.3390/ani12192645)
Supplement: Supplementary file 1 [file animals-12-02645-s001.zip › animals-1807568-supplementary.pdf]

Animas-1807568 Naughton et al. Association between socio-demographic factors and owners' beliefs and attitudes to pet cats fundamental dietary and physical exercise needs, in City of Belfast. Supplementary materials.

Table S1. Animals living environment and fundamental management practices, including, feeding practices, monitoring animal body weight and knowledge, and use of Body Condition Score (BCS) reported by the cat owners in this study. A full set of cross-tabulated results.

		Socio-demographic factors evaluated in the study															
	All respondents	*Effects of age*			*Effects of gender*			*Effects of occupation*		*Effects of education*				*Effects of employment*			
		18-25	*26-67*	*67+*	*[1]F*	*M*	*ND*	*NR*	*VA*	*1-4*	*5-6*	*7-8*	*ND*	*FT*	*PT*	*FTE*	*UR*
	n (%)	Cramer's V coefficient (C'sV)[2] *P* value[3] n (%)															
**I) Living environment provided to cats**		C'sV = 0.095 P = 0.130			C'sV = 0.107 P = 0.060			C'sV = 0.190 P < 0.001		C'sV = 0.092 P = 0.350				C'sV = 0.121 P = 0.071			
indoors (no access outdoors)	106(26.6)	46(43.4)	44(41.5)	16(15.1)	52(49.0)	50(47.2)	4(3.8)	79(74.5)	27(25.5)	57(53.7)	36(34.0)	9(8.5)	4(3.8)	63(59.4)	18(17.0)	7(6.6)	18(17.0)
indoors with a free access to outdoors	258(64.8)	78(30.2)	138(53.5)	42(16.3)	167(64.7)	85(33.0)	6(2.3)	232(89.9)	26(10.1)	130(50.4)	69(26.7)	39(15.1)	20(7.8)	107(41.4)	59(22.9)	25(9.7)	67(26.0)
outdoors with an access to shelter	34(8.5)	9(26.5)	18(52.9)	7(20.6)	17(50.0)	16(47.1)	1(2.9)	28(82.3)	6(17.7)	15(44.1)	11(32.3)	5(17.7)	2(5.9)	13(38.2)	7(20.6)	5(14.7)	9(26.5)
Total	398(100)	133(33.4)	200(50.2)	65(16.3)	236(59.3)	151(37.9)	11(2.8)	339(85.2)	59(14.8)	202(50.8)	116(29.1)	54(13.6)	26(6.5)	183(46.0)	84(21.1)	37(13.6)	94(9.3)
**II) Rationale behind daily feeding amount**		C'sV = 0.136 P = 0.144			C'sV = 0.206 P < 0.001			C'sV = 0.208 P = 0.004		C'sV = 0.129 P = 0.172				C'sV = 0.158 P = 0.012			
fed according to cat appetite	121(30.4)	38(31.4)	56(46.3)	27(22.3)	81(66.9)	38(31.4)	2(1.7)	110(90.9)	11(9.1)	71(58.7)	33(27.3)	9(7.4)	8(6.6)	56(46.3)	16(13.2)	10(8.3)	39(32.2)
following the advice from another person (non-professional)	47(11.8)	21(44.7)	20(42.5)	6(12.8)	21(44.7)	24(51.0)	2(4.3)	34(72.3)	13(27.7)	29(61.7)	12(25.5)	5(10.7)	1(2.1)	26(55.3)	13(27.7)	3(6.4)	5(10.6)
following veterinary advice	69(17.3)	20(29.0)	36(52.2)	13(18.8)	35(50.7)	32(46.4)	2(2.9)	58(84.1)	11(15.9)	32(46.4)	21(30.4)	12(17.4)	4(5.8)	32(46.4)	14(20.3)	5(7.2)	18(26.1)
following instructions on food packaging	121(30.4)	44(36.4)	65(53.7)	12(9.9)	79(65.3)	41(33.9)	1(0.8)	108(89.3)	13(10.7)	55(45.5)	36(29.7)	18(14.9)	12(9.9)	49(40.5)	37(30.6)	13(10.7)	22(18.2)
based on visual inspection of the animal behaviour	38(9.5)	9(23.7)	23(60.5)	6(15.8)	19(50.0)	16(42.1)	3(7.9)	27(71.1)	11(28.9)	14(36.9)	13(34.2)	10(26.3)	1(2.6)	20(52.6)	4(10.5)	5(13.2)	9(23.7)

Responses	Total	18-25	26-67	67+	F	M	ND	NR	VA	1-4	5-6	7-8	ND	FT	PT	FTE	UR
no answer provided	2(0.5)	1(50.0)	0(0.0)	1(50.0)	1(50.0)	0(0.0)	1(50.0)	2(100)	0(0.0)	1(50.0)	1(50.0)	0(0.0)	0(0.0)	0(0.0)	0(0.0)	1(50.0)	1(50.0)
Total	398(100)	133(33.4)	200(50.2)	65(16.3)	236(59.3)	151(37.9)	11(2.8)	339(85.2)	59(14.8)	202(50.8)	116(29.1)	54(13.6)	26(6.5)	183(46.0)	84(21.1)	37(13.6)	94(9.3)
**III) Frequency of monitoring animal body weight**		C'sV = 0.105 P = 0.067			C'sV = 0.122 P = 0.019			C'sV = 0.295 P < 0.001		C'sV = 0.156 P = 0.004				C'sV = 0.107 P = 0.164			
every 3-6 months	91(22.9)	35(38.4)	39(42.9)	17(18.7)	57(62.6)	29(31.9)	5(5.5)	60(65.9)	31(34.1)	46(50.5)	29(31.9)	15(16.5)	1(1.1)	49(53.8)	14(15.4)	8(8.8)	20(22.0)
once a year	116(29.1)	36(31.0)	69(59.5)	11(9.5)	78(67.2)	35(30.2)	3(2.6)	105(90.5)	11(9.5)	44(37.9)	43(37.1)	19(16.4)	10(8.6)	54(46.6)	28(24.1)	14(12.1)	20(17.2)
I don't monitor	191(48.0)	62(32.4)	92(48.2)	37(19.4)	101(52.9)	87(45.5)	3(1.6)	174(91.1)	17(8.9)	112(58.6)	44(23.0)	20(10.5)	15(7.9)	80(41.9)	42(22.0)	15(7.8)	54(28.3)
Total	398(100)	133(33.4)	200(50.2)	65(16.3)	236(59.3)	151(37.9)	11(2.8)	339(85.2)	59(14.8)	202(50.8)	116(29.1)	54(13.6)	26(6.5)	183(50.8)	84(29.1)	37(13.6)	94(6.5)
**IV) Body Condition Score (BCS): knowledge and use**		C'sV = 0.144 P = 0.036			C'sV = 0.174 P = 0.002			C'sV = 0.396 P < 0.001		C'sV = 0.141 P = 0.021				C'sV = 0.135 P = 0.040			
I know BCS and use it	65(16.3)	33(50.8)	21(32.3)	11(16.9)	42(64.6)	18(27.7)	5(7.7)	36 (55.4)	29(44.6)	22(33.9)	26(40.0)	16(24.6)	1(1.5)	33(50.8)	8(12.3)	13(20.0)	11(16.9)
I know of BCS, but not sure how to use it	74(18.6)	26(35.1)	35(47.3)	13(17.6)	32(43.2)	39(52.7)	3(4.1)	65 (87.8)	9(12.2)	40(54.1)	24(32.4)	7(9.5)	3(4.0)	38(51.4)	14(18.9)	8(10.8)	14(18.9)
I am vaguely familiar with BCS thus I do not use it	35(8.8)	9(25.7)	19(54.3)	7(20.0)	27(77.1)	8(22.9)	0(0.0)	31(88.6)	4(11.4)	21(60.0)	8(22.9)	5(14.2)	1(2.9)	12(34.3)	8(22.8)	3(8.6)	12(34.3)
I do not know and do not use BCS	224(56.3)	65(28.7)	125(56.0)	34(15.3)	135(60.5)	86(38.1)	3(1.4)	207(92.8)	17(7.2)	119(52.9)	58(26.0)	26(11.7)	21(9.4)	100(44.4)	54(24.2)	13(5.8)	57(25.6)
Total	398(100)	133(33.4)	200(50.2)	65(16.3)	236(59.3)	151(37.9)	11(2.8)	339(85.2)	59(14.8)	202(50.8)	116(29.1)	54(13.6)	26(6.5)	183(50.8)	84(29.1)	37(13.6)	94(6.5)

[1]F= Female; M=Male; ND=Not disclosed; NR=Not related to veterinary or animal sci.; VA= Veterinary, and animal sci. related; 1-4=ISCED 2011 level 1-4; 5-6=ISCED 2011 level 5-6; 7-8=ISCED 2011 level 7-8; ND=Not disclosed; FT=Employment status - Full-time; PT= Employment status - Part-time; FTE= Full time education; UR= Unemployed, retired, or otherwise at home and not working.

[2]Cramer's V coefficients (C'sV) show the level of associations between respondents' opinions and the sociodemographic ordinal variables listed in the table columns. C'sV between 0.100-0.150 indicate moderate while C'sV between 0.151-0.250 indicate strong association [17].

[3]P values for Pearson's chi-square tests or those for Fisher Freeman Hilton exact test for the data-subsets of smaller sample size according to Cochran condition.

Table S2. Owners attitude of cats dietary preferences, needs and the drivers of shopping for cat food. A full set of cross-tabulated results.

	Socio-demographic factors evaluated in the study															
Responses[1]	*Effects of age*			*Effects of gender*			*Effects of occupation*		*Effects of education*				*Effects of employment*			
	18-25	*26-67*	*67+*	[2]*F*	*M*	*ND*	*NR*	*VA*	*1-4*	*5-6*	*7-8*	*ND*	*FT*	*PT*	*FTE*	*UR*

			n (%)	Cramer's V coefficient (C′sV)[3] *P* value[4] n (%)				
**Owners perception of their animal(s) food preferences: I think that…**								
				C′sV = 0.091 P = 0.763	C′sV = 0.071 P = 0.673	C′sV = 0.130 P = 0.077	C′sV = 0.161 P = 0.306	C′sV = 0.175 P = 0.184
1	…my cat(s) are happier being fed human foods such as fresh meat or milk or table foods	NA	105(26.4)	39(37.1) 49(46.7) 17(16.2)	62(59.0) 40(38.1) 3(2.9)	83(79.1) 22(20.9)	42(40.0) 35(33.3) 19(18.1) 9(8.6)	56(53.3) 22(21.0) 8(7.6) 19(18.1)
		DF	77(19.3)	21(27.3) 43(55.8) 13(16.9)	41(53.2) 34(44.2) 2(2.6)	67(87.0) 10(13.0)	44(57.1) 19(24.7) 11(14.3) 3(3.9)	37(48.0) 17(22.1) 5(6.5) 18(23.4)
		VM	163(41.0)	57(34.9) 78(47.9) 28(17.2)	101(62.0) 56(34.3) 6(3.7)	139(85.3) 24(14.7)	87(53.4) 45(27.6) 21(12.9) 10(6.1)	66(40.5) 30(18.4) 22(13.5) 45(27.6)
		NS	53(13.3)	16(30.2) 30(56.6) 7(13.2)	32(60.4) 21(39.6) 0(0.0)	50(94.3) 3(5.7)	29(54.7) 17(32.1) 3(5.7) 4(7.5)	24(45.3) 15(28.3) 2(3.8) 12(22.6)
		Total	398(100)	133(33.4) 200(50.3) 65(16.3)	236(59.3) 151(37.9) 11(2.8)	339(85.2) 59(14.8)	202(50.8) 116(29.1) 54(13.6) 26(6.5)	183(46.0) 84(21.1) 37(13.6) 94(9.3)
				C′sV = 0.153 P = 0.147	C′sV = 0.075 P = 0.895	C′sV = 0.105 P = 0.221	C′sV = 0.133 P = 0.617	C′sV = 0.161 P = 0.307
2	…my cat(s) are happier being fed dry commercial food	NA	91(22.9)	35 (38.5) 42(46.1) 14(15.4)	59(64.8) 30(33.0) 2(2.2)	77(84.6) 14(15.4)	39(42.8) 28(30.8) 15(16.5) 9(9.9)	40(43.9) 22(24.2) 8(8.8) 21(23.1)
		DF	132(33.2)	52(39.4) 62(47.0) 18(13.6)	73(55.3) 55(41.7) 4((3.0)	110(83.3) 22(16.7)	69(52.3) 43(32.6) 13(9.8) 7(5.3)	64(48.5) 30(22.7) 16(12.1) 22(16.7)
		VM	130(32.7)	33(25.4) 75(57.7) 22(16.9)	78(60.0) 48(36.9) 4(3.1)	109(83.8) 21(16.2)	71(54.6) 33(25.4) 19(14.6) 7(5.4)	58(44.6) 24(18.5) 12(9.2) 36(27.7)
		NS	45(11.3)	13(28.9) 21(46.7) 11(24.4)	26(57.8) 18(40.0) 1(2.2)	43(95.6) 2(4.4)	23(51.1) 12(26.7) 7(15.5) 3(6.7)	21(46.7) 8(17.8) 1(2.2) 15(33.3)
		Total	398(100)	133(33.4) 200(50.3) 65(16.3)	236(59.3) 151(37.9) 11(2.8)	339(85.2) 59(14.8)	202(50.8) 116(29.1) 54(13.6) 26(6.5)	183(46.0) 84(21.1) 37(13.6) 94(9.3)
				C′sV = 0.095 P = 0.311	C′sV = 0.081 P = 0.517	C′sV = 0.115 P = 0.150	C′sV = 0.168 P = 0.239	C′sV = 0.159 P = 0.329
3	…my cat(s) are happier being fed wet commercial food	NA	41(10.63)	15 (36.6) 19(46.3) 7(17.1)	22(53.7) 18(43.9) 1(2.4)	33(80.5) 8(19.5)	22(53.7) 14(34.1) 1(2.4) 4(9.8)	21(51.2) 8(19.5) 4(9.8) 8(19.5)
		DF	86(21.6)	22(25.6) 44(51.2) 20(23.3)	44(51.1) 39(45.3) 3(3.5)	70(81.4) 16(18.6)	49(57.0) 23(26.8) 7(8.1) 7(8.1)	44(51.2) 14(16.3) 5(5.8) 23(26.7)
		VM	235(59.0)	86(36.6) 118(50.2) 31(13.2)	150(63.8) 79(33.6) 6(2.6)	208(88.5) 27(11.5)	112(47.7) 69(29.3) 40(17.0) 14(6.0)	104(44.2) 54(23.0) 27(11.5) 50(21.3)
		NS	36(9.0)	10(27.8) 19(52.8) 7(19.4)	20(55.5) 15(41.7) 1(2.8)	28(77.8) 8(22.2)	19(52.8) 10(27.8) 6(16.6) 1(2.8)	14(38.9) 8(22.2) 1(2.8) 13(36.1)
		Total	398(100)	133(33.4) 200(50.3) 65(16.3)	236(59.3) 151(37.9)11(2.8)	339(85.2) 59(14.8)	202(50.8) 116(29.1) 54(13.6) 26(6.5)	183(46.0) 84(21.1) 37(13.6) 94(9.3)
**Owners perception of an expression of care towards their animal(s): I think that…**								

				C’sV = 0.094	C’sV = 0.101	C’sV = 0.143	C’sV = 0.084	C’sV = 0.093
				P = 0.734	P = 0.225	P = 0.043	P = 0.499	P = 0.323
1	…my cat(s) want food when it approaches me e.g., with tail up or brushing against me	NA	73(18.3)	24(32.9) 40(54.8) 9(12.3)	51(69.9) 20(27.4) 2(2.7)	56(76.7) 17(23.3)	30(41.1) 24(32.9) 15(20.5) 4(5.5)	41(56.2) 12(16.4) 7(9.6) 13(17.8)
		DF	84(21.1)	30(35.7) 37(44.1) 17(20.2)	43(51.2) 38(45.2) 3(3.6)	72(85.7) 12(14.3)	46(54.8) 24(28.6) 9(10.7) 5(5.9)	38(45.3) 20(23.8) 9(10.7) 17(20.2)
		VM	171(43.0)	59(34.5) 86(50.3) 26(15.2)	98(57.3) 70(40.9) 3(1.8)	154(90.1) 17(9.9)	87(50.9) 52(30.4) 22(12.9) 10(5.8)	71(41.5) 38(22.2) 19(11.1) 43(25.2)
		NS	70(17.6)	20(28.6) 37(52.9) 13 (18.6)	44(62.9) 23(32.9) 3(4.2)	57(81.4) 13(18.6)	39(55.7) 16(22.9) 8(11.4) 7(10.0)	33(47.1) 14(20.0) 2(2.9) 21(30.0)
		Total	398(100)	133(33.4) 200(50.3) 65(16.3)	236(59.3) 151(37.9) 11(2.8)	339(85.2) 59(14.8)	202(50.8) 116(29.1) 54(13.6) 26(6.5)	183(46.0) 84(21.1) 37(13.6) 94(9.3)
				C’sV = 0.087	C’sV = 0.108	C’sV = 0.091	C’sV = 0.102	C’sV = 0.072
				P = 0.427	P = 0.158	P = 0.351	P = 0.186	P = 0.720
2	…it is important to give food/treats to my cat(s) to show how much I care	NA	111(27.9)	32(28.8) 55(49.6) 24(21.6)	66(59.5) 42(37.8) 3(2.7)	89(80.2) 22(19.8)	45(40.6) 35(31.5) 19(17.1) 12(10.8)	47(42.4) 22(19.8) 13(11.7) 29(26.1)
		DF	114(28.6)	35(30.7) 62(54.4) 17(14.9)	63(55.2) 50(43.9) 1(0.9)	98(86.0) 16(14.0)	69(60.5) 27(23.7) 14(12.3) 4(3.5)	53(46.5) 29(25.5) 7(6.1) 25(21.9)
		VM	127(31.9)	49(38.6) 59(46.4) 19(15.0)	75(59.1) 45(35.4) 7(5.5)	112(88.2) 15(11.8)	64(50.4) 40(31.5) 16(12.6) 7(5.5)	57(44.9) 26(20.5) 13(10.2) 31(24.4)
		NS	46(11.6)	17(37.0) 24(52.2) 5(10.8)	32(69.6) 14(30.4) 0(0.0)	40(87.0) 6(13.0)	24(52.2) 14(30.4) 5(10.9) 3(6.5)	26(56.5) 7(15.2) 4(8.7) 9(19.6)
		Total	398(100)	133(33.4) 200(50.3) 65(16.3)	236(59.3) 151(37.9) 11(2.8)	339(85.2) 59(14.8)	202(50.8) 116(29.1) 54(13.6) 26(6.5)	183(46.0) 84(21.1) 37(13.6) 94(9.3)
	**Owners perception of their animal(s) dietary needs: I think that…**							
				C’sV = 0.154	C’sV = 0.246	C’sV = 0.240	C’sV = 0.166	C’sV = 0.060
				P = 0.138	P < 0.001	P < 0.001	P = 0.253	P = 0.886
1	…food wise, my cat(s) gets what it needs for its age/body condition and health	NA	20(5.0)	7(35.0) 7(35.0) 6(30.0)	14(70.0) 6(30.0) 0(0.0)	11(55.0) 9(45.0)	8(40.0) 7(35.0) 3(15.0) 2(10.0)	11(55.0) 3(15.0) 2(10.0) 4(20.0)
		DF	39(9.8)	19(48.7) 14(35.9) 6(15.4)	11(28.2) 27(69.2) 1(2.6)	28(71.8) 11(28.2)	20(51.3) 15(38.5) 0(0.0) 4(10.2)	21(53.8) 9(23.1) 1(2.6) 8(20.5)
		VM	309(77.6)	96(31.1) 166(53.7) 47(15.2)	192(62.1) 110(35.6) 7(2.3)	274(88.7) 35(11.3)	156(50.5) 89(28.8) 46(14.9) 18(5.8)	136(44.0) 66(21.4) 31(10.0) 76(24.6)
		NS	30(7.5)	11(36.7) 13(43.3) 6(20.0)	19(63.3) 8(26.7) 3(10.0)	26(86.7) 4(13.3)	18(60.0) 5(16.7) 5(16.7) 2(6.6)	15(50.0) 6(20.0) 3(10.0) 6(20.0)
		Total	398(100)	133(33.4) 200(50.3) 65(16.3)	236(59.3) 151(37.9) 11(2.8)	339(85.2) 59(14.8)	202(50.8) 116(29.1) 54(13.6) 26(6.5)	183(46.0) 84(21.1) 37(13.6) 94(9.3)
				C’sV = 0.094	C’sV = 0.079	C’sV = 0.099	C’sV = 0.081	C’sV = 0.128
				P = 0.317	P = 0.540	P = 0.273	P = 0.550	P = 0.020
2	…my cat(s) are being fed by neighbours or is/are hunting, so	NA	109(27.4)	42(38.5) 51(46.8) 16(14.7)	65(59.6) 41(37.6) 3(2.8)	93(85.3) 16(14.7)	54(49.5) 31(28.5) 19(17.4) 5(4.6)	52(47.7) 32(29.3) 10(9.2) 15(13.8)
		DF	102(25.6)	31(30.4) 49(48.0) 22(21.6)	57(55.9) 43(42.1) 2(2.0)	83(81.4) 19(18.6)	54(52.9) 29(28.4) 13(12.8) 6(5.9)	51(50.0) 12(11.8) 9(8.8) 30(29.4)

	they are getting more food than	VM	105(26.4)	39(37.2) 52(49.5) 14(13.3)	59(56.2) 41(39.0) 5(4.8)	88(83.8) 17(16.2)	47(44.8) 35(33.3) 12(11.4) 11(10.5)	44(41.9) 25(23.8) 13(12.4) 23(21.9)
	I give them	NS	82(20.6)	21(25.6) 48(58.5) 13(15.9)	55(67.1) 26(31.7) 1(1.2)	75(91.5) 7(8.5)	47(57.3) 21(25.6) 10(12.2) 4(4.9)	36(43.9) 15(18.3) 5(6.1) 26(31.7)
		Total	398(100)	133(33.4) 200(50.3) 65(16.3)	236(59.3) 151(37.9) 11(2.8)	339(85.2) 59(14.8)	202(50.8) 116(29.1) 54(13.6) 26(6.5)	183(46.0) 84(21.1) 37(13.6) 94(9.3)
**Owners motivation regarding their choices of purchasing commercial food for their cat(s): I buy cat food primarily …**								
				C’sV = 0.094	C’sV = 0.113	C’sV = 0.048	C’sV = 0.105	C’sV = 0.072
				P = 0.324	P = 0.115	P = 0.823	P = 0.161	P = 0.722
1	…considering price	NA	167(42.0)	47(28.1) 91(54.5) 29(17.4)	109(65.3) 56(33.5) 2(1.2)	144(86.2) 23(13.8)	69(41.3) 54(32.3) 31(18.6) 13(7.8)	83(49.7) 34(20.3) 13(7.8) 37(22.2)
		DF	96(24.1)	32(33.3) 51(53.1) 13(13.6)	50(52.1) 44(45.8) 2(2.1)	81(84.4) 15(15.6)	55(57.3) 27(28.1) 10(10.4) 4(4.2)	43(44.8) 23(24.0) 10(10.4) 20(20.8)
		VM	107(26.9	42(39.2) 45(42.1) 20(18.7)	63(58.9) 38(35.5) 6(5.6)	89(83.2) 18(16.8)	61(57.0) 29(27.1) 10(9.4) 7(6.5)	45(42.1) 22(20.5) 9(8.4) 31(29.0)
		NS	28(7.0)	12(42.9) 13(46.4) 3(10.7)	14(50.0) 13(46.4) 1(3.6)	25(89.3) 3(10.7)	17(60.7) 6(21.4) 3(10.7) 2(7.2)	12(42.8) 5(17.9) 5(17.9) 6(21.4)
		Total	398(100)	133(33.4) 200(50.3) 65(16.3)	236(59.3) 151(37.9) 11(2.8)	339(85.2) 59(14.8)	202(50.8) 116(29.1) 54(13.6) 26(6.5)	183(46.0) 84(21.1) 37(13.6) 94(9.3)
				C’sV = 0.148	C’sV = 0.244 P < 0.001	C’sV = 0.175	C’sV =0.146	C’sV = 0.099
				P = 0.008		P = 0.006	P = 0.466	P = 0.915
2	…considering what (s)he likes	NA	58(14.6)	17(29.3) 28(48.3) 13(22.4)	31(53.4) 24(31.4) 3(5.2)	48(82.8) 10(17.2)	27(46.6) 19(32.8) 6(10.3) 6(10.3)	29(50.0) 9(15.5) 5(8.6) 15(25.9)
	to eat	DF	68(17.1)	31(45.6) 23(33.8) 14(20.6)	28(41.2) 35(51.5) 5(7.3)	53(77.9) 15(22.1)	37(54.4) 22(32.4) 7(10.3) 2(2.9)	30(44.1) 17(25.0) 4(5.9) 17(25.0)
		VM	247(62.1)	72(29.1) 138(55.9) 37(15.0)	166(67.2) 78(31.6) 3(1.2)	221(89.5) 26(10.5)	123(49.8) 67(27.1) 39(15.8) 18(7.3)	111(44.9) 53(21.5) 25(10.1) 58(23.5)
		NS	25(6.3)	13(52.0) 11(44.0) 1(4.0)	11(44.0) 14(56.0) 0(0.0)	17(68.0) 8(32.0)	15(60.0) 8(32.0) 2(8.0) 0(0.0)	13(52.0) 5(20.0) 3(12.0) 4(16.0)
		Total	398(100)	133(33.4) 200(50.3) 65(16.3)	236(59.3) 151(37.9) 11(2.8)	339(85.2) 59(14.8)	202(50.8) 116(29.1) 54(13.6) 26(6.5)	183(46.0) 84(21.1) 37(13.6) 94(9.3)
				C’sV = 0.051	C’sV = 0.102	C’sV = 0.125	C’sV = 0.112	C’sV = 0.116
				P = 0.910	P = 0.222	P = 0.103	P = 0.096	P = 0.067
3	…considering what is available	NA	145(36.4)	47(32.4) 77(53.1)21(14.5)	84(57.9) 60(41.4) 1(0.7)	122(84.1) 23(15.9)	60(41.4) 46(31.7) 26(17.9) 13(9.0)	75(51.7) 30(20.7) 11(7.6) 29(20.0)
	in the shops I go to	DF	114(28.6)	37(32.5) 59(51.7) 18(15.8)	70(61.4) 41(36.0) 3(2.6)	91(79.8) 23(20.2)	57(50.0) 34(29.8) 16(14.1) 7(6.1)	53(49.4) 24(21.1) 13(11.4) 24(21.1)
		VM	122(30.7)	42(34.4) 57(46.7) 23(18.9)	73(59.9) 42(34.4) 7(5.7)	110(90.2) 12(9.8)	75(61.5) 32(26.2) 9(7.4) 6(4.9)	44(36.1) 30(24.6) 10(8.2) 38(31.1)
		NS	17(4.3)	7(41.2) 7(41.2) 3(17.6)	9(52.9) 8(47.1) 0(0.0)	16(94.1) 1(5.9)	10(58.8) 4(23.5) 3(17.7) 0(0.0)	11(64.8) 0(0.0) 3(17.6) 3(17.6)
		Total	398(100)	133(33.4) 200(50.3) 65(16.3))	236(59.3) 151(37.9) 11(2.8)	339(85.2) 59(14.8)	202(50.8) 116(29.1) 54(13.6) 26(6.5)	183(46.0) 84(21.1) 37(13.6) 94(9.3)

				C’sV = 0.175<br>P<0.001	C’sV = 0.202<br>P = 0.009	C’sV = 0.171<br>P = 0.007	C’sV = 0.134<br>P = 0.608	C’sV = 0.149<br>P = 0.466
4	…considering the health of my cat	NA	68(17.1)	30(44.2) 19(27.9) 19(27.9)	29(42.7) 36(52.9) 3(4.4)	49(72.1) 19(27.9)	30(44.1) 23(33.8) 9(13.3) 6(8.8)	36(52.9) 15(22.1) 2(2.9) 15(22.1)
		DF	62(15.6)	27(43.5) 29(46.8) 6(9.7)	33(53.2) 26(41.9) 3(4.9)	56(90.3) 6(9.7)	32(51.6) 21(33.9) 7(11.3) 2(3.2)	26(42.0) 10(16.1) 10(16.1) 16(25.8)
		VM	232(58.3)	64(27.6) 131(56.5) 37(15.9)	155(66.8) 72(31.0) 5(2.2)	204(87.9) 28(12.1)	119(51.3) 62(26.7) 33(14.2) 18(7.8)	103(44.4) 51(22.0) 23(9.9) 55(23.7)
		NS	36(9.0)	12(33.3) 21(58.4) 3(8.3)	19(52.8) 17(47.2) 0(0.0)	30(83.3) 6(16.7)	21(58.3) 10(27.8) 5(13.9) 0(0.0)	18(50.0) 8(22.2) 2(5.6) 8(22.2)
		Total	398(100)	133(33.4) 200(50.3) 65(16.3)	236(59.3) 151(37.9) 11(2.8)	339(85.2) 59(14.8)	202(50.8) 116(29.1) 54(13.6) 26(6.5)	183(46.0) 84(21.1) 37(13.6) 94(9.3)

[1] NA=Not at all; DF=Doubt-fully; VM=Very much so; NS=Not sure; [2]Cramer’s V coefficients (C’sV) show the level of associations between respondents’ opinions and the sociodemographic ordinal variables listed in the table columns. C’sV between 0.100-0.150 indicate moderate while C’sV between 0.151-0.250 indicate strong association [17].

[2]F= Female; M=Male; ND=Not disclosed; NR=Not related to veterinary or animal sci.; VA= Veterinary, and animal sci. related; 1-4=ISCED 2011 level 1-4; 5-6=ISCED 2011 level 5-6; 7-8=ISCED 2011 level 7-8; ND=Not disclosed; FT=Employment status - Full-time; PT= Employment status - Part-time; FTE= Full time education; UR= Unemployed, retired, or otherwise at home and not working.

[3] P values for Pearson’s chi-square tests or those for Fisher Freeman Hilton exact test for the data-subsets of smaller sample size according to Cochran’s condition.

Table S3. Owners’ perceptions of their cats’ needs and satisfaction. *A full set of cross-tabulated results.*

		Responses[1]	*Socio-demographic factors evaluated in the study*				
			Effects of age	*Effects of gender*	*Effects of occupation*	*Effects of education*	*Effects of employment*
			18-25 26-67 67+	*[2]F M ND*	*NR VA*	*1-4 5-6 7-8 ND*	*FT PT FTE UR*
		n (%)	Cramer's V coefficient (C’sV)[3]<br>*P* value[4]<br>n (%)				
**Owners perception of their animal ability to self-regulate**							
			C’sV = 0.133<br>P = 0.714	C’sV = 0.149<br>P = 0.062	C’sV = 0.208<br>P = 0.004	C’sV = 0.108<br>P = 0.532	C’sV = 0.102<br>P = 0.652
1	Pet cats can get all the exercise they need themselves	SA 84 (21.1)	21(25.0) 45 (53.6) 18(21.4)	51(60.7) 29 (34.5) 4(4.8)	73(86.9) 11(13.1)	41(48.8) 25 (29.8) 14(16.7) 4(4.7)	35(41.7) 16(19.0) 5(6.0) 28(33.3)
		A 142(35.7)	46(32.4) 76(53.5) 20(14.1)	96(67.6) 43(30.3) 3(2.1)	131(92.2) 11(7.8)	75(52.8) 37(26.0) 16(11.3) 14(9.9)	63(44.4) 33(23.2) 16(11.3) 30(21.1)
		N 77(19.3)	32(41.5) 33(42.9) 12(15.6)	36(46.7) 40(52.0) 1(1.3)	59(76.6) 18(23.4)	41(53.2) 25(32.5) 9(11.7) 2(2.6)	40(52.0) 14(18.2) 6(7.8) 17(22.0)
		SD 33(8.3)	13(39.4) 15(45.4) 5(15.2)	18(54.5) 15(45.5) 0(0.0)	23(69.7) 10(30.3)	12(36.4) 14(42.4) 5(15.1) 2(6.1)	18(54.6) 7(21.2) 4(12.1) 4(12.1)

		D 41(10.3	14(34.1) 20(48.8) 7(17.1)	23(56.1) 15(36.6) 3(7.3)	34(82.9) 7(17.1)	19(46.4) 11(26.8) 8(19.5) 3(7.3)	18(43.9) 10(24.4) 5(12.2) 8(19.5)
		NS 21(5.3)	7(33.3) 11(52.4) 3(14.3)	12(57.1) 9(42.9) 0(0.0)	19(90.5) 2(9.5)	14(66.7) 4(19.0) 2(9.5) 1(4.8)	9(42.9) 4(19.0) 1(4.8) 7(33.3)
		Total 398(100)	133(33.4) 200(50.3) 65(16.3)	236(59.3) 151(37.9) 11(2.8)	339(85.2) 59(14.8)	202(50.8) 116(29.1) 53(13.6) 26(6.5)	183(46.0) 84(21.1) 37(9.3) 94(23,6)
			C′sV = 0.138	C′sV = 0.135	C′sV = 0.176	C′sV = 0.120	C′sV = 0.119
			P = 0.125	P = 0.688	P = 0.031	P = 0.310	P = 0.317
2	Pet cats can regulate their own physical activity /daily exercise to keep healthy	SA 49(12.3)	14(28.6) 24 (49.0) 11(22.4)	36(73.5) 11(22.4) 2(4.1)	38(77.5) 11(22.5)	23(47.0) 13(26.5) 10(20.4) 3(6.1)	20(40.8) 10(20.4) 4(8.2) 15(30.6)
		A 154(38.7)	47(30.5) 83(53.9) 24(15.6)	87(56.5) 62(40.3) 5(3.2)	140(90.9) 14(9.1)	81(52.6) 43(27.9) 14(9.1) 16(10.4)	64(41.5) 38(24.7) 12(7.8) 40(26.0)
		N 92(23.1)	29(31.5) 49(53.3) 14(15.2)	51(55.4) 39(42.4) 2(2.2)	77(83.7) 15(16.3)	48(52.2) 30(32.6) 11(12.0) 3(3.2)	46(50.0) 17(18.5) 10(10.8) 19(20.7)
		SD 39(9.8)	19(48.7) 12(30.8) 8(20.5)	22(56.4) 16(41.0) 1(2.6)	31(79.5) 8(20.5)	21(53.8) 11(28.2) 6(15.4) 1(2.6)	23(59.0) 6(15.4) 3(7.7) 7(17.9)
		D 49(12.3)	22(44.9) 21(42.9) 6(12.2)	31(63.3) 17(34.7) 1(2.0)	38(77.5) 11(22.5)	23(46.9) 14(28.6) 11(22.5) 1(2.0)	22(44.9) 12(24.5) 8(16.3) 7(14.3)
		NS 15(3.8)	2(13.3) 11(73.34) 2(13.3)	9(60.0) 6(40.0) 0(0.0)	15(100) 0(0.0)	6(40.0) 5(33.4) 2(13.3) 2(13.3)	8(53.3) 1(6.7) 0(0.0) 6(40.0)
		Total 398(100	133(33.4) 200(50.3) 65(16.3))	236(59.3) 151(37.9) 11(2.8)	339(85.2) 59(14.8)	202(50.8) 116(29.1) 53(13.6) 26(6.5)	183(46.0) 84(21.1) 37(9.3) 94(23,6))
			C′sV = 0.118	C′sV = 0.127	C′sV = 0.137	C′sV = 0.119	C′sV = 0.102
			P = 0.349	P = 0.232	P = 0.191	P = 0.317	P = 0.647
3	Pet cats can regulate themselves on how much they need to eat daily	SA 30(7.5)	7(23.3) 15(50.0) 8(26.7)	20(66.7) 8(26.6) 2(6.7)	23(76.7) 7(23.3)	13(43.3) 9(30.0) 5(16.7) 3(10.0)	16(53.3) 4(13.3) 0(0.0) 10(33.4)
		A 130(32.7)	37(28.5) 74(56.9) 19(14.6)	73(56.1) 56(43.1) 1(0.8)	116(89.2) 14(10.8)	78(60.0) 34(26.1) 10(7.7) 8(6.2)	63(48.5) 27(20.8) 11(8.4) 29(22.3)
		N 67(16.8)	29(43.3) 28(41.8) 10(14.9)	37(55.2) 28(41.8) 2(3.0)	55(82.1) 12(17.9)	35(52.2) 21(31.3) 8(12.0) 3(4.5)	29(43.3) 18(26.9) 7(10.4) 13(19.4)
		SD 68(17.1)	27(39.7) 30(44.1) 11(16.2)	46(67.6) 18(26.5) 4(5.9)	55(80.9) 13(19.1)	25(36.8) 24(35.3) 14(20.6) 5(7.3)	31(45.6) 15(22.1) 6(8.8) 16(23.5)
		D 77(19.3)	26(33.8) 37(48.0) 14(18.2)	44(57.1) 31(40.3) 2(2.6)	65(84.4) 12(15.6)	35(45.4) 23(29.9) 14(18.2) 5(6.5)	31(40.2) 15(19.5) 12(15.6) 19(24.7)
		NS 26(6.5)	7(26.9) 16(61.5) 3(11.6)	16(61.5) 10(38.5) 0(0.0)	25(96.2) 1(3.8)	16(61.6) 5(19.2) 3(11.5) 2(7.7)	13(50.0) 5(19.2) 1(3.9) 7(26.9)
		Total 398(100	133(33.4) 200(50.3) 65(16.3)	236(59.3) 151(37.9) 11(2.8)	339(85.2) 59(14.8)	202(50.8) 116(29.1) 53(13.6) 26(6.5)	183(46.0) 84(21.1) 37(9.3) 94(23,6)
**Owners perception of cats physical activity needs**							
			C′sV = 0.097	C′sV = 0.128	C′sV = 0.145	C′sV = 0.126	C′sV = 0.148
			P = 0.676	P = 0.227	P = 0.126	P = 0.218	P = 0.036
1							

	Pet cats need to be kept active by their owner to keep them fit	SA 28(11.6)	5(17.9) 18(64.2) 5(17.9)	15(53.6) 11(39.3) 2(7.1)	22(78.6) 6(21.4)	15(53.6) 5 (17.9) 7(25.0) 1(3.5)	12(42.9) 3(10.7) 4(14.3) 9(32.1)
		A 143(26.6)	50(35.0) 70(48.9) 23(16.1)	76(53.1) 61(42.7) 6(4.2)	118(82.5) 25(17.5)	69(28.2) 51(35.7) 18(12.6) 5(3.5)	82(57.3) 20(14.0) 13(9.1) 28(19.6)
		N 104(19.3)	39(37.5) 52(50.0) 13(12.5)	63(60.6) 38(36.5) 3(2.9)	85(81.7) 19(18.3)	51(49.0) 32(30.8) 14(13.5) 7(6.7)	42(40.4) 26(25.0) 13(12.5) 23(22.1)
		SD 36(11.6)	11(30.6) 17(47.2) 8(22.2)	20(55.6) 16(44.4) 0(0.0)	33(91.7) 3(8.3)	16(44.5) 12(33.3) 3(8.3) 5(13.9)	16(44.4) 9(25.0) 4(11.1) 7(19.5)
		D 80(26.4)	27(33.7) 38(47.5) 15(18.8)	57(71.2) 23(28.8) 0(0.0)	75(93.7) 5(6.3)	47(58.7) 15(18.7) 11(13.8) 7(8.8)	29(36.2) 23(28.8) 3(3.8) 25(31.2)
		NS 7(4.5)	1(14.3) 5(71.4) 1(14.3)	5(71.4) 2(28.6) 0(0.0)	6(85.7) 1(14.3)	4(57.1) 1(14.3) 1(14.3) 1(14.3)	2(28.6) 3(42.9) 0(0.0) 2(28.5)
		Total 398(100)	133(33.4) 200(50.3) 65(16.3)	236(59.3) 151(37.9) 11(2.8)	339(85.2) 59(14.8)	202(50.8) 116(29.1) 53(13.6) 26(6.5)	183(46.0) 84(21.1) 37(9.3) 94(23,6)
			C′sV = 0.147	C′sV = 0.081	C′sV = 0.078	C′sV = 0.130	C′sV = 0.137
			P = 0.072	P = 0.871	P = 0.790	P = 0.166	P = 0.097
2	Pet cats need to be provided with less physical exercise as compare to pet dogs	SA 43(10.8)	16(37.2) 21(48.8) 6(14.0)	21(48.8) 21(48.8) 1(2.4)	34(79.1) 9(20.9)	21(48.8) 14(32.6) 4(9.3) 4(9.3)	18(41.8) 9(21.0) 4(9.3) 12(27.9)
		A 157(39.4)	51(32.5) 74(47.1) 32(20.4)	94(59.9) 58(36.9) 5(3.2)	137(87.3) 20(12.7)	86(54.8) 41(26.1) 17(10.8) 13(8.3)	67(42.7) 29(18.5) 14(8.9) 47(29.9)
		N 63(15.8)	17(27.0) 36(57.1) 10(15.9)	42(66.7) 19(30.1) 2(3.2)	52(82.5) 11(17.5)	27(42.9) 26(41.2) 7(11.1) 3(4.8)	33(52.4) 11(17.5) 7(11.1) 12(19.0)
		SD 37(9.3)	14(37.8) 18(48.7) 5(13.5)	23(62.2) 14(37.8) 0(0.0)	31(83.8) 6(16.2)	19(51.4) 10(27.0) 8(21.6) 0(0.0)	22(59.5) 9(24.3) 2(5.4) 4(10.8)
		D 69(17.3)	29(42.0) 37(53.6) 3(4.4)	39(56.5) 28(40.6) 2(2.9)	60(87.0) 9(13.0)	38(55.1) 16(23.2) 13(18.8) 2(2.9)	34(49.3) 18(26.1) 9(13.0) 8(11.6)
		NS 29(7.3)	6(20.7) 14(48.3) 9(31.0)	17(58.6) 11(37.9) 1(3.5)	25(86.2) 4(13.8)	11(37.9) 9(31.0) 5(17.3) 4(13.8)	9(31.0) 8(27.6) 1(3.5) 11(37.9)
		Total 398(100)	133(33.4) 200(50.3) 65(16.3)	236(59.3) 151(37.9) 11(2.8)	339(85.2) 59(14.8)	202(50.8) 116(29.1) 53(13.6) 26(6.5)	183(46.0) 84(21.1) 37(9.3) 94(23,6)
			C′sV = 0.103	C′sV = 0.114	C′sV = 0.074	C′sV = 0.100	C′sV = 0.129
			P = 0.589	P = 0.413	P = 0.825	P = 0.675	P = 0.173
3	It is difficult to get pet cats to exercise	SA 46(11.6)	13(28.2) 24(52.2) 9(19.6)	26(56.5) 20(43.5) 0(0.0)	40(87.0) 6(13.0)	22(47.8) 17(37.0) 5(10.9) 2(4.3)	19(41.3) 8(17.4) 2(4.3) 17(37.0)
		A 106(26.6)	42(39.6) 46(43.4) 18(17.0)	57(53.8) 47(44.3) 2(1.9)	91(85.8) 15(14.2)	57(53.8) 28(26.4) 12(11.3) 9(8.5)	47(44.3) 20(18.9) 14(13.2) 25(23.6)
		N 77(19.3)	31(40.3) 36(46.7) 10(13.00)	49(63.6) 23(29.9) 5(6.5)	67(87.0) 10(13.0)	38(49.3) 24(31.2) 10(13.0) 5(6.5)	36(46.7) 14(18.2) 12(15.6) 15(19.5)
		SD 46(11.6)	11(23.9) 28(60.9) 7(15.2)	28(60.8) 17(37.0) 10(2.2)	36(78.3) 10(21.4)	25(54.4) 14(30.4) 6(13.0) 1(2.2)	27(58.7) 10(21.7) 1(2.2) 8(17.4)
		D 105(26.4)	31(29.5) 57(54.3) 15(16.2)	65(61.9) 38(36.2) 2(1.9)	90(85.7) 15(14.3)	53(50.5) 30(28.6) 16(15.2) 6(5.7)	47(44.7) 28(26.7) 7(6.7) 23(21.9)
		NS 18(4.5)	5(27.8) 9(50.0) 4(22.2)	11(61.1) 6(33.3) 1(5.6)	15(83.3) 3(16.7)	7(38.9) 3(16.7) 5(27.7) 3(16.7)	7(38.9) 4(22.2) 1(5.6) 6(33.3)
		Total 398(100)	133(33.4) 200(50.3) 65(16.3)	236(59.3) 151(37.9) 11(2.8)	339(85.2) 59(14.8)	202(50.8) 116(29.1) 53(13.6) 26(6.5)	183(46.0) 84(21.1) 37(9.3) 94(23,6)

			C′sV = 0.118<br>P = 0.354	C′sV = 0.190<br>P = 0.001	C′sV = 0.169<br>P = 0.045	C′sV = 0. 164<br>P = 0.006	C′sV = 0.129<br>P = 0.176
4	The amount of exercise a pet cat needs depends on its age, body condition and medical condition	SA 174(43.7)	64(36.8) 78(44.8) 32(18.4)	100(57.5) 68(39.1) 6(3.4)	137(78.7) 37(21.3)	76(43.7) 68(39.1) 21(12.0) 9(5.2)	87(50.0) 28(16.1) 21(12.1) 38(21.8)
		A 164(41.2)	46(28.1) 94(57.3) 24(14.6)	95(57.9) 68(41.5) 1(0.6)	148(90.2) 16(9.8)	92(56.1) 42(25.6) 19(11.6) 11(6.7)	76(46.4) 34(20.7) 10(6.1) 44(26.8)
		N 24(6.0)	7(29.2) 13(54.2) 4(16.6)	19(79.2) 5(20.8) 0(0.0)	22(91.7) 2(8.3)	17(70.9) 2(8.3) 3(12.5) 2(8.3)	8(33.3) 10(41.7) 2(8.3) 4(16.7)
		SD 8(2.0)	4(50.0) 3(37.5) 1(12.5)	3(37.5) 4(50.0) 1(12.5)	7(87.5) 1(12.5)	3(37.5) 2(25.0) 2(25.0) 1(12.5)	3(37.5) 3(37.5) 1(12.5) 1(12.5)
		D 10(2.5)	6(60.0) 4(40.0) 0(0.0)	9(90.0) 1(10.0) 0(0.0)	8(80.0) 2(20.0)	6(60.0) 1(10.0) 2(20.0) 1(10.0)	4(40.0) 3(30.0) 0(0.0) 3(30.0)
		NS 18(4.5)	6(33.3) 8(44.5) 4(22.2)	10(55.6) 5(27.8) 3(16.7)	17(94.4) 1(5.6)	8(44.4) 1(5.6) 7(38.9) 2(11.1)	5(27.8) 6(33.3) 3(16.7) 4(22.2)
		Total 398(100	133(33.4) 200(50.3) 65(16.3)	236(59.3) 151(37.9) 11(2.8)	339(85.2) 59(14.8)	202(50.8) 116(29.1) 53(13.6) 26(6.5)	183(46.0) 84(21.1) 37(9.3) 94(23,6)

Owners perception of selected environmental factors effects on their cats

			C′sV = 0.110<br>P = 0.475	C′sV = 0.155<br>P = 0.038	C′sV =0.188<br>P = 0.015	C′sV = 0.188<br>P = 0.015	C′sV = 0.117<br>P = 0.364
1	Keeping a few cats together can assure that each cat is well exercised	SA 26(6.5)	9(34.6) 10(38.5) 7(26.9)	20(76.9) 5(19.2) 1(3.9)	23(88.5) 3(11.5)	12(46.1) 8(30.8) 4(15.4) 2(7.7)	5(19.2) 6(23.1) 4(15.4) 11(42.3)
		A 105(26.4)	34(32.4) 53(50.5) 18(17.1)	51(48.6) 50(47.6) 4(3.8)	90(85.7) 15(14.3)	51(48.6) 35(33.3) 14(13.3) 5(4.8)	54(51.4) 19(18.1) 10(9.5) 22(21.0)
		N 95(23.9)	30(31.6) 49(51.6) 16(16.8)	57(60.0) 38(40.0) 0(0.0)	85(89.5) 10(10.5)	55(57.9) 25(26.3) 10(10.5) 5(5.3)	44(46.3) 25(26.3) 6(6.3) 20(21.1)
		SD 24(6.0)	11(45.8) 11(45.8) 2(8.4)	13(54.2) 11(45.8) 0(0.0)	15(62.5) 9(37.5)	14(58.3) 6(25.0) 4(16.7) 0(0.0)	13(54.1) 6(25.0) 1(4.2) 4(16.7)
		D 64(16.1)	27(42.2) 30(46.9) 7(10.9)	46(71.9) 15(23.4) 3(4.7)	51(79.7) 13(20.3)	33(51.6) 22(34.4) 5(7.8) 4(6.2)	29(45.3) 13(20.3) 8(12.5) 14(21.9)
		NS 84(21.1)	22(26.2) 47(55.9) 15(17.9)	49(58.3) 32(38.1) 3(3.6)	75(89.3) 9(10.7)	37(44.1) 20(23.8) 17(20.2) 10(11.9)	38(45.2) 15(17.9) 8(9.5) 23(27.4)
		Total 398(100)	133(33.4) 200(50.3) 65(16.3)	236(59.3) 151(37.9) 11(2.8)	339(85.2) 59(14.8)	202(50.8) 116(29.1) 53(13.6) 26(6.5)	183(46.0) 84(21.1) 37(9.3) 94(23,6)
			C′sV = 0.092<br>P = 0.745	C′sV = 0.108<br>P = 0.510	C′sV = 0.087<br>P = 0.710	C′sV = 0.151<br>P = 0.026	C′sV = 0.097<br>P = 0.734
2	Keeping a few cats together can lead to stress to some cats	SA 45(6.5)	18(40.0) 19(42.2) 8(17.8)	34(75.6) 10(22.2) 1(2.2)	37(82.2) 8(17.8)	20(44.5) 14(31.1) 10(22.2) 1(2.2)	22(48.9) 10(22.2) 3(6.7) 10(22.2)
		A 152(26.4)	51(33.5) 81(53.3) 20(13.2)	92(60.5) 57(37.5) 3(2.0)	129(84.9) 23(15.1)	73(48.0) 53(34.9) 16(10.5) 10(6.6)	65(42.8) 34(22.4) 18(11.8) 35(23.0)
		N 70(23.9)	24(34.3) 36(51.4) 10(14.3)	35(50.0) 33(47.1) 2(2.9)	62(88.6) 8(11.4)	41(58.6) 17(24.3) 8(11.4) 4(5.7)	39(55.8) 12(17.1) 7(10.0) 12(17.1)

		SD 24(6.0)	8(33.3) 12(50.0) 4(16.7)	13(54.2) 10(41.7) 1(4.2)	21(87.5) 3(12.5)	8(33.4) 11(45.8) 5(20.8) 0(0.0)	13(54.2) 5(20.8) 1(4.2) 5(20.8)
		D 30(16.1)	12(40.0) 12(40.0) 6(20.0)	19(63.4) 10(33.3) 1(3.3)	23(76.7) 7(23.3)	18(60.0) 9(30.0) 1(3.3) 2(6.7)	10(33.4) 7(23.3) 4(13.3) 9(30.0)
		NS 77(21.1)	20(26.0) 40(51.9) 17(22.1)	43(55.8) 31(40.3) 3(3.9)	67(87.0) 10(13.0)	42(54.5) 12(15.6) 14(18.2) 9(11.7)	34(14.1) 16(20.8) 4(5.2) 23(29.9)
		Total 398(100)	133(33.4) 200(50.3) 65(16.3)	236(59.3) 151(37.9) 11(2.8)	339(85.2) 59(14.8)	202(50.8) 116(29.1) 53(13.6) 26(6.5)	183(46.0) 84(21.1) 37(9.3) 94(23.6)
			C′sV = 0.134 P = 0.157	C′sV = 0.118 P = 0.348	C′sV = 0.240 P < 0.001	C′sV = 0.129 P = 0.173	C′sV = 0.121 P = 0.288
3	Keeping cat(s) with pet dogs can assure that pet cats are well exercised	SA 27(6.8)	11(40.8) 7(25.9) 9(33.3)	11(40.7) 15(55.6) 1(3.7)	16(59.3) 11(40.7)	12(44.5) 10(37.0) 3(11.1) 2(7.4)	10(37.1) 6(22.2) 2(7.4) 9(33.3)
		A 79(19.8)	29(36.7) 39(49.4) 11(13.9)	46(58.2) 30(38.0) 3(3.8)	65(82.3) 14(17.7)	48(60.7) 24(30.4) 6(7.6) 1(1.3)	44(55.7) 12(15.2) 10(12.7) 13(16.4)
		N 99(24.9)	34(34.4) 53(53.5) 12(12.1)	59(59.6) 37(37.4) 3(3.0)	91(91.9) 8(8.1)	49(49.5) 26(26.3) 13(13.1) 11(11.1)	46(46.4) 27(27.3) 8(8.1) 18(18.2)
		SD 27(6.8)	8(29.6) 15(55.6) 4(14.8)	12(44.4) 15(55.6) 0(0.0)	23(85.2) 4(14.8)	9(33.3) 11(40.8) 4(14.8) 3(11.1)	14(51.9) 3(11.1) 1(3.7) 9(33.3)
		D 71(17.8)	27(38.0) 34(47.9) 10(14.1)	48(67.6) 22(31.0) 1(1.4)	57(80.3) 14(19.7)	35(49.3) 23(32.4) 9(12.7) 4(5.6)	26(36.6) 16(22.5) 9(12.7) 20(28.2)
		NS 95(23.9)	24(25.3) 52(54.7) 19(20.0)	60(63.1) 32(33.7) 3(3.2)	87(91.6) 8(8.4)	49(51.6) 22(23.2) 19(20.0) 5(5.3)	43(45.3) 20(21.0) 7(7.4) 25(26.3)
		Total 398(100)	133(33.4) 200(50.3) 65(16.3)	236(59.3) 151(37.9) 11(2.8)	339(85.2) 59(14.8)	202(50.8) 116(29.1) 53(13.6) 26(6.5)	183(46.0) 84(21.1) 37(9.3) 94(23,6)
			C′sV = 0.188 P = 0.002	C′sV = 0.090 P = 0.774	C′sV = 0.169 P = 0.045	C′sV = 0.169 P = 0.004	C′sV = 0.160 P = 0.010
4	Pet cat(s) are happier being kept outdoors	SA 22 (5.5)	7(31.8) 10 (45.5) 5(22.7)	15(68.2) 6(27.3) 1(4.5)	17(77.3) 5(22.7)	11(50.0) 8(36.4) 3(13.6) 0(0.0)	7(31.8) 3(13.6) 4(18.2) 8(36.4)
		A 93(23.4)	36(38.7) 45(48.4) 12(12.9)	55(59.1) 34(36.6) 4(4.3)	72(77.4) 21(22.6)	38(40.9) 38(40.9) 13(13.9) 4(4.3)	46(49.5) 21(22.6) 11(11.8) 15(16.1)
		N 128(32.2)	40(31.3) 74(57.8) 14(10.9)	69(53.9) 57(44.5) 2(1.6)	115(89.8) 13(10.2)	71(55.5) 35(27.3) 15(11.7) 7(5.5)	67(52.3) 27(21.1) 10(7.8) 24(18.8)
		SD 52(13.1)	26(50.0) 18(34.6) 8(15.4)	32(61.5) 18(34.6) 2(3.9)	42(80.8) 10(19.2)	24(46.2) 18(34.6) 6(11.5) 4(7.7)	22(42.3) 11(21.1) 7(13.5) 12(23.1)
		D 84(21.1)	15(17.9) 48(57.1) 21(25.0)	52(61.9) 30(35.7) 2(2.4)	77(91.7) 7(8.3)	51(60.7) 15(17.9) 9(10.7) 9(10.7)	38(45.2) 13(15.5) 5(6.0) 28(33.3)
		NS 19(4.8)	9(47.4) 5(26.3) 5(26.3)	13(68.4) 6(31.6) 0(0.0)	16(84.2) 3(15.8)	7(36.9) 2(10.5) 8(42.1) 2(10.5)	3(15.8) 9(47.4) 0(0.0) 7(36.8)
		Total 398(100)	133(33.4) 200(50.3) 65(16.3)	236(59.3) 151(37.9) 11(2.8)	339(85.2) 59(14.8)	202(50.8) 116(29.1) 53(13.6) 26(6.5)	183(46.0) 84(21.1) 37(9.3) 94(23,6)
			C′sV = 0.094 P = 0.715	C′sV = 0.077 P = 0.912	C′sV =0.065 P = 0.891	C′sV = 0.104 P = 0.608	C′sV = 0.102 P = 0.647

5	Pet cat(s) are happier being kept indoors	SA 26(6.5)	10(38.5)	12 (46.1)	4(13.4)	18(69.2)	7(26.9)	1(3.9)	22(84.6)	4(15.4)	14(53.8)	8(30.8)	2(7.7)	2(7.7)	11(42.3)	6(23.1)	3(11.5)	6(23.1)
		A 101(25.4)	37(36.6)	48(47.5)	16(15.9)	62(61.4)	36(35.6)	3(3.0)	83(82.2)	18(17.8)	48(47.5)	36(35.6)	10(9.9)	7(6.9)	51(50.5)	22(21.8)	10(9.9)	18(17.8)
		N 136(34.2)	40(29.4)	75(55.1)	21(15.4)	80(58.8)	53(39.0)	3(2.2)	117(86.0)	19(14.0)	74(54.4)	38(27.9)	17(12.5)	7(5.2)	66(48.5)	25(18.4)	13(9.6)	32(23.5)
		SD 49(12.3)	20(40.8)	20(40.8)	9(18.4)	24(49.0)	23(46.9)	2(4.1)	44(89.8)	5(102)	26(53.1)	10(20.4)	8(16.3)	5(10.2)	19(38.8)	10(20.4)	5(10.2)	15(30.6)
		D 71(17.8)	20(28.2)	40(56.3)	11(15.5)	42(59.2)	27(38.0)	2(2.8)	60(84.5)	11(15.5)	34(47.9)	21(29.6)	12(16.9)	4(5.6)	33(46.5)	15(21.1)	6(8.5)	17(23.9)
		NS 15(3.8)	6(40.0)	5(33.3)	4(26.7)	10(66.7)	5(33.3)	0(0.0)	13(86.7)	2(13.3)	6(40.0)	3(20.0)	5(33.3)	1(6.7)	3(20.0)	6(40.0)	0(0.0)	6(40.0)
		Total 398(100)	133(33.4)	200(50.3)	65(16.3)	236(59.3)	151(37.9)	11(2.8)	339(85.2)	59(14.8)	202(50.8)	116(29.1)	53(13.6)	26(6.5)	183(46.0)	84(21.1)	37(9.3)	94(23.6)

[1] SA=Strongly agree, A=Agree, N=Neutral, SA=Strongly disagree, D=Disagree, NS=Not sure.

[2]F= Female; M=Male; ND=Not disclosed; NR=Not related to veterinary or animal sci.; VA= Veterinary, and animal sci. related; 1-4=ISCED 2011 level 1-4; 5-6=ISCED 2011 level 5-6; 7-8=ISCED 2011 level 7-8; ND=Not disclosed; FT=Employment status - Full-time; PT= Employment status - Part-time; FTE= Full time education; UR= Unemployed, retired, or otherwise at home and not working.

[3]Cramer's V coefficients (C'sV) show the level of associations between respondents' opinions and the sociodemographic ordinal variables listed in the table columns. C'sV between 0.100-0.150 indicate moderate while C'sV between 0.151-0.250 indicate strong association [17].

[4]P values for Pearson's chi-square tests or those for Fisher Freeman Hilton exact test for the data-subsets of smaller sample size according to Cochran's condition.
